# Supplementary material for: Opportunities for better value wound care: a multiservice, cross-sectional survey of complex wounds and their care in a UK community population
Source: BMJ Open. 2018 Mar 22;8(3):e019440. doi: 10.1136/bmjopen-2017-019440 (PMC5875675; doi:10.1136/bmjopen-2017-019440)
Supplement: Supplementary data [file bmjopen-2017-019440supp001.pdf]

Office use only: ID number

☐☐☐☐

MANCHESTER 1824
The University of Manchester

NHS
National Institute for Health Research
Collaboration for Leadership in Applied Health Research and Care (CLAHRC) Greater Manchester

A Survey of Complex Wounds and their Care in the Community

Patient Data Capture Form

Date this form was completed: ☐☐ / ☐☐ / ☐☐☐☐
day *month* *year*

This form is to be completed by the NHS health care professional (from the participating NHS community-based services) delivering most of the hands on wound care for a person with one or more complex wounds. <u>Please aim to ensure that only one form is completed per patient</u>

Section 1:

1.1 What is your main clinical role currently? *(Please cross (x) one option)*

District Nurse/community nurse ☐ Nurse Specialist ☐

Podiatrist ☐ Other ☐

If 'Other' please state: ☐

1.2 What is your NHS Agenda for Change band (as the health care professional from the participating NHS community-based services delivering most of the hands on wound care for this patient)? *(Please cross (x) one option)*

Band 3 ☐ Band 4 ☐ Band 5 ☐ Band 6 ☐ Band 7 ☐ Band 8a ☐

Other ☐ If 'Other' please state: ☐

1.3 Currently, in which location does this patient receive <u>most</u> of their hands on wound care? *(Please cross (x) one option)*

Patient's home ☐ Community clinic/heath centre/GP practice ☐ Nursing home ☐

Residential home ☐ Hospice ☐ Prison ☐

Other ☐ If 'Other' please state: ☐

1.4 How many wound related consultations (with all professionals involved) has THIS patient had in the last seven days?: ☐☐

1.5 Duration of the most recent consultation: ☐☐☐ minutes

1.6 Are any other health professionals involved in deciding or planning wound care for this person? ***(Please cross (x) one option)***

Yes ☐   **N**o ☐   Don't know ☐

↓

1.7 If Yes who are they? ***(please cross (x) all that apply)*****:**

GP ☐   Tissue viability nurse ☐   Other wound care specialist ☐

Podiatrist ☐   District nurse ☐

Other ☐   If 'Other' please state: ☐

Section 2: Patient details

2.1 Patient's DOB: dd ☐☐ mm ☐☐ yyyy ☐☐☐☐

2.2 Patient's gender: male ☐ female ☐

2.3 Ethnicity:

White British ☐   White Irish ☐   White Other ☐   Black African ☐   Black Caribbean ☐   Black Other ☐

Asian Indian ☐   Asian Pakistani ☐   Asian Bangladeshi ☐   Asian Other ☐   White and Black Caribbean ☐   White and Black African ☐

White and Asian ☐   Other mixed background ☐   Chinese ☐   Other ☐

2.4 Please state location of patient's GP ***(Please cross (x) one option)***

Central Manchester ☐   South Manchester ☐   Salford ☐   Oldham ☐

Bury ☐   Heywood, Middleton, Rochdale ☐   Trafford ☐

Other location ☐   If other, please state: ☐

Don't know ☐

2.5 What type of accommodation best describes where this patient currently lives? ***(Please cross (x) one option)***

Own / rented home ☐ Nursing home ☐ Residential home ☐

No fixed abode ☐ Prison ☐

Other ☐ If 'Other' please state: ☐

2.6 What co-morbidities does this patient have? ***(please cross (x) all that apply)***:

**Cardiovascular disease (CVD)**	e.g. Hypertension (high blood pressure, high BP), Myocardial infarction (MI, heart attack, IHD), Angina (IHD), Heart failure (CCF, CHF, HF, LVF)	☐
**Peripheral Arterial Disease**	e.g. Ischaemia (leg/foot), Claudication (intermittent claudication), Rest pain, Arteriopathy/angiopathy, Aneurysm including aortic aneurysm	☐
**Diabetes (DM)**	e.g. Insulin dependent DM (IDDM, Type I diabetes), Non insulin dependent DM (NIDDM, Type II diabetes)	☐
**Arthritis**	e.g. Rheumatoid arthritis (RA), Osteoarthritis (OA), Non rheumatoid arthritis	☐
**Orthopaedics**	e.g. Fractured neck of femur (# NOF, # hip, fractured hip, broken hip), Other fracture/break, Elective orthopaedic	☐
**Airways**	e.g. Chronic obstructive airways/pulmonary disease (COAD, COPD), Chronic bronchitis, Emphysema, Asthma	☐
**Neurological**	e.g. Parkinson's disease, Multiple sclerosis (MS), Epilepsy (fits, Grand mal, Petit mal, tonic-clonic, seizure), Spinal Injury	☐
**Stroke**	e.g. (cerebrovascular accident, CVA, transient ischaemic attack, TIA)	☐
**Cancer**	e.g. any cancer diagnosis	☐

2.7 Patient's Continence ***(please cross (x) all that apply)***:

No incontinence ☐ Urinary incontinence ☐ Faecal Incontinence ☐

2.8 Patient mobility ***(please cross (x) one option)***:

Patient walks freely ☐ Patient walks with difficultly ☐ Patient is immobile ☐

2.9 In your judgement is this patient at risk of pressure ulceration? ***(please cross (x) one option):***

Yes ☐ No ☐ Don't know ☐

↓

2.10 If yes is their pressure ulcer risk documented in their notes/care plan? ***(please cross (x) one option)*****:**

Yes ☐ No ☐ Don't know ☐

Section 3: Current complex wounds

3.1 Please record the number of wounds (by wound type) that this patient has.

**Wound type:**		**Number of wound of this type (please circle one number for each type or leave blank if 0):**							
**Foot ulcer in patient with diabetes**	**RIGHT foot**	1	2	3	4	5	6	7	8 or more
	**LEFT foot**	1	2	3	4	5	6	7	8 or more
**Foot ulcer in patient without diabetes**	**RIGHT foot**	1	2	3	4	5	6	7	8 or more
	**LEFT foot**	1	2	3	4	5	6	7	8 or more
**Venous leg ulcer**	**RIGHT leg**	1	2	3	4	5	6	7	8 or more
	**LEFT leg**	1	2	3	4	5	6	7	8 or more
**Arterial leg ulcer**	**RIGHT leg**	1	2	3	4	5	6	7	8 or more
	**LEFT leg**	1	2	3	4	5	6	7	8 or more
**Arterial/venous (mixed aetiology) leg ulcer**	**RIGHT leg**	1	2	3	4	5	6	7	8 or more
	**LEFT leg**	1	2	3	4	5	6	7	8 or more
**Pressure ulcer**		1	2	3	4	5	6	7	8 or more

**Dehisced surgical wound currently healing by secondary intention**	1	2	3	4	5	6	7	8 or more
**Wound resulting from excision of pilonidal sinus**	1	2	3	4	5	6	7	8 or more
**Wound resulting from excision of peri-anal abscess**	1	2	3	4	5	6	7	8 or more
**Other surgical wound healing by secondary intention**	1	2	3	4	5	6	7	8 or more
**Traumatic wound (e.g. result of road traffic accident)**	1	2	3	4	5	6	7	8 or more
**Fungating carcinoma**	1	2	3	4	5	6	7	8 or more
**Burn**	1	2	3	4	5	6	7	8 or more

**Other wound type - please describe:**	**Number of wound of this type (please circle):**							
	1	2	3	4	5	6	7	8 or more
	1	2	3	4	5	6	7	8 or more
	1	2	3	4	5	6	7	8 or more

Section 4: Treatment of complex wounds

We now want to collect information on the treatment of this patient's complex wound(s). If the patient has more than one complex wound (of any type) please now focus on the one wound you consider to be the worst/most severe (if there are more than one wound of equal severity choose any one of these wounds and complete the following information for just this wound).

We will refer to the wound for which the following data are collected as the selected wound.

4.1 Please record what type of wound the selected wound is (as noted above - this is the most severe/wound and therefore the one you are focusing on in this section). ***Please cross (x) one option.***

- Foot ulcer in patient with diabetes ☐
- Foot ulcer in patient without diabetes ☐
- Venous leg ulcer ☐
- Arterial leg ulcer ☐
- Arterial/venous leg ulcer ☐
- Pressure ulcer ☐
- Dehisced surgical wound currently healing by secondary intention ☐
- Wound resulting from excision of pilonidal sinus ☐
- Wound resulting from excision of peri-anal abscess ☐
- Other surgical wound healing by secondary intention ☐
- Traumatic wound (e.g. result of road traffic accident) ☐
- Fungating carcinoma ☐
- Burn ☐
- Other wound type ☐ If 'Other' please state: ______

4.2 Please note the location/area most affected by the selected wound. ***Please cross (x) one option.***

head/neck ☐	arm ☐	hand ☐	breast ☐
abdomen ☐	back ☐	sacrum ☐	natal cleft ☐
buttock ☐	peri-anal ☐	perineum ☐	leg ☐
foot ☐	Other ☐	If 'Other' please state: ______	

Please record the following details for the selected wound. ***Please cross (x) one option.***

4.3 Exudate level:

High ☐ Moderate/ medium ☐ Low ☐ None ☐

4.4 Malodour:

Yes ☐ No ☐

4.5 For the <u>selected wound</u>, which wound dressings are being used currently? ***(Refer to the list of dressings in the guidelines and insert the corresponding code in each of the boxes)*****:**

Primary dressing ☐☐ Secondary dressing ☐☐

Please record any other <u>wound treatments</u> for the <u>selected wound:</u>

4.6 Drugs/medicines: ***(please cross (x) all that apply)***

Antibiotics for wound infections ☐

Pentoxifylline ☐

Topical steroids ☐

Analgesics ☐

Other ☐ If 'Other' please state: ☐

4.7 For the <u>selected wound</u>, the bandage currently used: (*Please cross (x) one option).*

N/A ☐

4 layer compression bandage ☐

Short stretch bandage (e.g. Actico/Comprilan) ☐

3 layer reduced compression bandage ☐

2 layer compression bandage (e.g. K2) ☐

Non-compression bandage (plus wadding) ☐

Dressing retention bandage (e.g. K-band) ☐

Other ☐ If 'Other' please state: ☐

4.8 Hosiery (stockings) for the leg affected by the <u>selected wound:</u> (*Please cross (x) one option).*

N/A ☐

Standard class 1 ☐

Standard class 2 ☐

Standard class 3 ☐

Made to measure Class 1 ☐

Made to measure Class 2 ☐

Made to measure Class 3 ☐

Standard two layer compression hosiery (aiming to deliver 40mmhg) ☐

Made to measure two layer compression hosiery (aiming to deliver 40mmhg) ☐

Other ☐ If 'Other' please state: ☐

Please record any equipment used relating to the <u>selected wound:</u>

4.9 Pressure relief and mobility: ***(please cross (x) all that apply)***

Pressure relieving	**mattresses:**	**cushions:**
High specification foam replacement e.g. MSS gilde	☐	☐
Air alternating replacement e.g. Quattro deep cell prime	☐	☐
Air alternating overlay e.g. Alpha excel	☐	☐
Repose (overlay system)	☐	☐
Foam overlay	☐	☐

Crutches ☐

Wheelchair ☐

Other ☐

If 'Other' please state: ____________

4.10 Footwear/Orthotics/Foot pressure reliving equipment: ***(please cross (x) all that apply)***

Prescribed footwear ☐

Footwear adaptation ☐

Insoles ☐

Heel pressure relief ☐
e.g. Repose Heel Trough, PROFO boot

Other ☐ If 'Other' please state: ____________

4.11 Other therapies for the <u>selected wound:</u> ***(please cross (x) all that apply)***

Hyperbaric oxygen therapy ☐

Physiotherapy ☐

Occupational therapy ☐

Negative pressure wound therapy ☐

Other ☐ If 'Other' please state: ____________

4.12 Treatment objectives for the selected wound: ***(please cross (x) all that apply)***

Debridement ☐ Absorption (removal of exudate) ☐

Hydration (keep the wound moist) ☐ Microbial load management ☐

Odour Management ☐ Reduce overgranulation ☐

Encourage granulation ☐ Protection ☐

Other ☐ If 'other' please state: ☐

4.13 If the selected wound is a PRESSURE ULCER record its category here (otherwise leave blank). (*Please cross (x) one option).*

I ☐ II ☐ III ☐ IV ☐ Unstageable ☐ Not applicable ☐

If the selected wound is a LEG ULCER please record the following (otherwise leave blank):

4.14 Is a Doppler ABPI for the leg with the selected wound recorded in the notes? (*Please cross (x) one option).*

Yes ☐ No ☐ Don't know ☐

4.15 If yes – please note the ABPI measure here:

☐☐•☐☐
