# Supplementary material for: Opportunities for better value wound care: a multiservice, cross-sectional survey of complex wounds and their care in a UK community population
Source: BMJ Open. 2018 Mar 22;8(3):e019440. doi: 10.1136/bmjopen-2017-019440 (PMC5875675; doi:10.1136/bmjopen-2017-019440)
Supplement: Author's manuscript [file bmjopen-2017-019440.draft_revisions.pdf]

BMJ Open

BMJ Open is committed to open peer review. As part of this commitment we make the peer review history of every article we publish publicly available.

When an article is published we post the peer reviewers' comments and the authors' responses online. We also post the versions of the paper that were used during peer review. These are the versions that the peer review comments apply to.

The versions of the paper that follow are the versions that were submitted during the peer review process. They are not the versions of record or the final published versions. They should not be cited or distributed as the published version of this manuscript.

BMJ Open is an open access journal and the full, final, typeset and author-corrected version of record of the manuscript is available on our site with no access controls, subscription charges or pay-per-view fees (http://bmjopen.bmj.com).

If you have any questions on BMJ Open's open peer review process please email editorial.bmjopen@bmj.com

BMJ Open

OPPORTUNITIES FOR BETTER VALUE WOUND CARE: investigating the prevalence and management of complex wounds in a UK community population

Journal:	*BMJ Open*
Manuscript ID	bmjopen-2017-019440
Article Type:	Research
Date Submitted by the Author:	01-Sep-2017
Complete List of Authors:	Gray, Trish; University of Manchester, Division of Nursing, Midwifery and Social Work, School of Health Sciences, Faculty of Biology, Medicine and Health; NIHR CLAHRC Greater Manchester<br>Rhodes, Sarah; University of Manchester, Centre for Biostatistics, Faculty of Biology, Medicine and Health; NIHR CLAHRC Greater Manchester<br>Atkinson, Ross; University of Manchester, Division of Nursing, Midwifery and Social Work, School of Health Sciences, Faculty of Biology, Medicine and Health; NIHR CLAHRC Greater Manchester<br>Rothwell, Katy; NIHR CLAHRC Greater Manchester, 3rd Floor Mayo Building, Salford Royal NHS Foundation Trust<br>Wilson, Paul; University of Manchester, Alliance Manchester Business School<br>Dumville, Jo C.; University of Manchester, Division of Nursing, Midwifery and Social Work, School of Health Sciences, Faculty of Biology, Medicine and Health; NIHR CLAHRC Greater Manchester<br>Cullum, Nicky; The University of Manchester, Division of Nursing, Midwifery and Social Work, School of Health Sciences, Faculty of Biology, Medicine and Health; NIHR CLAHRC Greater Manchester
**Primary Subject Heading**:	Health services research
Secondary Subject Heading:	Evidence based practice
Keywords:	WOUND MANAGEMENT, Healthcare value, Prevalence survey, Patient centred care, Patient safety, Healthcare quality

SCHOLARONE™
Manuscripts

OPPORTUNITIES FOR BETTER VALUE WOUND CARE: investigating the prevalence and management of complex wounds in a UK community population

Corresponding Author

Trish Gray
Division of Nursing, Midwifery and Social Work; School of Health Sciences;
Faculty of Biology, Medicine and Health.
The University of Manchester, Room 6.332, Jean McFarlane Building,
Oxford Road, Manchester, M13 9PL.
Email: Trish.Gray@manchester.ac.uk
Tel: (+44) (0)161 306 7849

Authors

Trish A Gray,[1,4] Sarah Rhodes,[2,4] Ross A Atkinson,[1,4] Katy Rothwell,[4] Paul Wilson,[3,4] Jo C Dumville,[1,4] Nicky A Cullum.[1,4,5]

Author Affiliations

1 Division of Nursing, Midwifery and Social Work, School of Health Sciences, Faculty of Biology, Medicine and Health, University of Manchester, Manchester, UK

2 Centre for Biostatistics, Faculty of Biology, Medicine and Health, University of Manchester, Manchester, UK

3 Alliance Manchester Business School, University of Manchester, Manchester, UK

4 NIHR CLAHRC Greater Manchester 3rd Floor Mayo Building, Salford Royal NHS Foundation Trust, Stott Lane, Salford, Manchester, UK

5 Research and Innovation Division, Central Manchester University Hospitals NHS Foundation Trust, Grafton Street, Manchester, UK

Word Count

Abstract: 285

Manuscript: 3935

For peer review only

Key Words

Health services research, Evidence based practice, Wound management, Healthcare value, Healthcare quality, Prevalence survey, Patient-centred care, Patient safety

Abstract

Background

Complex wounds impose a substantial health economic burden worldwide. As wound care is managed across multiple settings by a range of healthcare professionals with varying levels of expertise, the actual care delivered can vary considerably and result in the underuse of evidence-based interventions, the overuse of interventions supported by limited evidence and low value healthcare.

Objectives

To quantify the number, type and management of complex wounds being treated over a two-week period of time and to explore variations in care by comparing current practices in wound assessment, prevention and treatment

Design

A multi-service cross-sectional survey

Setting

This survey spanned eight community services within five Northern England NHS Trusts

Results

The point prevalence of complex wounds in this community based population was 16.4 per 10,000 (95% confidence interval 15.9 to 17.0). Based on data from 3179 patients, antimicrobial dressings were being used as the primary dressing for 36% of patients with complex wounds. Forty percent of people with leg ulcers either had not received the recommended Doppler-aided ABPI assessment, or it was unclear whether a

For peer review only

recording had been taken. Thirty-one percent of patients whose most severe wound was a venous leg ulcer were not receiving compression therapy and there was limited use of two-layer compression hosiery. Of patients with a pressure ulcer, 39% were not using a pressure relieving cushion or mattress.

Conclusions

Marked variations were found in care, underuse of evidence-based practices and overuse of practices that are not supported by robust research evidence. Significant opportunities for delivering better value wound care therefore exist. Efforts should now focus on developing strategies to identify, assess and disinvest from products and practices supported by little or no evidence, and enhance the uptake of those that are.

Article Summary

Strengths and limitations of this study

- This cross-sectional survey provides robust community-focused population point-prevalence estimates for different types of complex wounds.
- This is the first community-focused multi-service survey to capture the wide variation in treatment and care of complex wounds between different NHS trusts.
- The survey is based on wound care provided by community services and may have missed patients only treated by other services providers such as acute or primary care or those self-treating.
- As this was an anonymised survey we were unable to neither conduct any case validation nor validate wound aetiology.

For peer review only

For peer review only - http://bmjopen.bmj.com/site/about/guidelines.xhtml

Introduction

Complex wounds, such as leg, foot and pressure ulcers which heal by secondary intention, impose a substantial health economic burden worldwide. In the UK, the point prevalence of complex wounds is estimated at 14.7 per 10,000 population, suggesting that approximately 80,000 people in the UK have one or more complex wound at any one time.[1] Whilst the annual cost of managing wounds has been crudely estimated at around £3 billion in the UK,[2] $2.85 billion in Australia[3] and $25 billion in the US,[4] the true cost is unknown. The increasing prevalence of complex wounds with age[5] and multimorbidity[6] means that it is difficult to separate the cost of wound care from the cost of caring for people with complex needs.

Wound care is managed across multiple settings by a range of healthcare professionals (HCPs) with varying levels of expertise.[7-11] HCPs are constantly under pressure to make the right choices for their patients, but when faced with an array of wound care products to choose from,[12] inaccessible or limited research evidence to guide decisions[7-12] and silo-based cost-control measures,[13] decisions may not be based on best practice[14 15] and fragmentation of practice and services may occur.[16] With such diversity, it is unlikely that all patients with wounds will have access to good value healthcare.[17] Unwarranted variation in healthcare at a time of rising demand is a concern for health systems globally. It has been estimated that around a third of medical practices are effective or likely to be effective, 50% are of unknown effectiveness and 15% are harmful or unlikely to be beneficial.[18] The cost-effectiveness[19] and value are even less well known.

Value in healthcare is centred on the interests and activities of all stakeholders and is measured by outcomes achieved relative to cost. This perspective shifts the focus on the delivery of care from volume alone to the value gained for patients from the healthcare investments being made e.g. staff time and treatment costs.[17] Every individual should have access to value-based care that considers their needs, preferences and priorities.[17] Gaining maximum healthcare value, given available resources, has been central to discussions and policy changes worldwide in recent years.[20] Decision makers, especially those at policy and organisational levels, are increasingly aiming to maximise patient benefit whilst minimising the opportunity costs of current healthcare approaches. The term "opportunity cost" refers to the potential for utilised

For peer review only - http://bmjopen.bmj.com/site/about/guidelines.xhtml

resources to achieve more value elsewhere in the healthcare system.[21] High opportunity costs occur with the overuse of ineffective treatments (or those that make a very small clinical difference), leading to wasted patient benefit and reduced value in the healthcare system. Conversely, underuse of treatments known to be effective also leads to waste.[20]

In some cases, large improvements in the value of healthcare can be made relatively easily by the implementation of evidence based guidelines[12 22 23] and smarter procurement of services and products.[24] We are seeing the development of initiatives focussed on reducing variation and improving value, not only in the UK,[21 24-26] but across the world.[27-29] One campaign, 'Choosing Wisely' was launched in 2012 and has been adopted by 12 countries.[30]

The first step to reducing unwarranted variation is to identify: 1) overuse of interventions that do not clearly offer value and 2) underuse of interventions known to offer value. Once such situations have been identified, organisations can start to work towards increased use of effective practices across the relevant populations they are responsible for. To date there have been no such initiatives focused in wound care; a significant yet often neglected area of care and healthcare spend, where there is great potential for better value care. To this end we explored variation in common interventions for complex wounds across eight different community services (spanning five NHS Trusts) in the North of England. This project forms part of a wider wound care programme developed by the National Institute for Health Research Collaboration for Leadership in Applied Health Research and Care Greater Manchester (NIHR CLAHRC GM). Given the dearth of basic epidemiological data on chronic or complex wounds in community settings,[31-33] we sought to quantify the number, type and management of complex wounds being treated over a two-week period of time. We then compared current practices in wound assessment, prevention and treatment with evidence-based recommendations, allowing exploration of variations in care between areas where distinct healthcare organisations provide care to geographically proximal residents. Key treatments of interest included the use of antimicrobial wound dressings, compression for people with venous leg ulcers (VLU) and the use of pressure ulcer relief for pressure ulcer prevention, as these are specifically highlighted in recent guidelines;

For peer review only - http://bmjopen.bmj.com/site/about/guidelines.xhtml

National Institute for Health and Clinical Excellence,[12 22 34] Scottish Intercollegiate Guidelines Network[35] and Health Technology Assessment,[36] as presented in Box 1.

Box 1. Key evidence and recommendations related to the assessment, prevention and treatment of complex wounds

Wound	Key treatments of interest	Guidelines and Recommendations
**Infected (All complex wounds)**	Silver dressings	Insufficient evidence to support the use of silver-containing dressings to promote wound healing or prevent wound infections.[12]
	Honey	Some high quality evidence (based on 2 RCTs only) has shown honey to heal partial thickness burns and infected post-operative wounds more quickly than comparators, however, comparators may not be relevant to current practice. Insufficient evidence to support the use of honey in other wounds.[12]
	Iodine	Iodine has been found to be superior to some non-antiseptic dressings but inferior to local antibiotic therapy. However, much of evidence is out of date and there is insufficient evidence addressing effectiveness and safety for use of iodine to treat or prevent wound infection.[12]
**Venous leg ulcer (VLU)**	Ankle Brachial Pressure Index (ABPI)	Measurement of ABPI should be performed by appropriately trained practitioners to substantiate the presence or absence of peripheral arterial disease (PAD) at initial assessment and to regularly review the use of compression therapy[35]
	Compression therapy	Simple non-adherent dressings and high compression multicomponent bandaging should be used for treating VLU patients with ABPI ≥ 0.8. Graduated compression hosiery is recommended to prevent recurrence of VLU.[35]
		Two layer compression stockings are as clinically effective as high compression bandages but more cost effective[33]
	Pentoxifylline	High quality evidence has found improved VLU healing with the use of pentoxifylline (believed to increase microcirculatory blood flow although exact mechanism of action is unknown) and should be considered in patients with VLU.[35]
**Diabetic foot ulcer**	Dressing choice	Insufficient evidence to support the use of any specific dressing. Clinical assessment and patient preference should be taken into consideration whilst the lowest acquisition cost appropriate to the clinical circumstances should be used.[12 34]
	Pressure relief	Offer non-removable casting to offload plantar neuropathic, non-ischaemic, uninfected forefoot and midfoot diabetic ulcers taking into consideration clinical assessment and patient preference. Use pressure redistributing devices and strategies to minimise the risk of pressure ulcers developing.[34]
**Pressure ulcer (PU)**	Dressing choice	Insufficient evidence to support the use of any specific dressing, choice should be determined by the patient's pain, tolerance, location of the ulcer and amount of exudate. A dressing that promotes a warm, moist wound healing environment should be considered for grade 2, 3 and 4 Pus.[12 22]
	Pressure relief	Use high-specification foam mattresses, or consider the use of dynamic support surface if not sufficient. Consider high specification foam or equivalent pressure redistributing cushion for chair or wheelchair use.[22]

For peer review only

For peer review only - http://bmjopen.bmj.com/site/about/guidelines.xhtml

Materials and Methods

Project design and participating organisations

A multi-service cross-sectional survey recorded wound prevalence and care for people living with complex wounds across eight community services within five NHS Trusts in the north of England (with a population of 1.9 million). The methods used here were based on a previous multi-service complex wound survey undertaken in Leeds, UK.[1]

As most wound care takes place in the community, the project focused on community-based wound care and did not include hospitals or primary care. HCPs working in a range of services (including tissue viability, adult and children's community nursing, podiatry, intermediate care, burns and plastics, specialist leg ulcer and specialist diabetic foot teams,) collected data for consecutive patients over a period of two weeks at each site between June 2015 and September 2016. Face-to-face training and accompanying instructions were provided to improve data accuracy and conformity of responses. Reminders were provided via telephone and email in the week leading to commencement. All services had a helpline number they could call if they had any questions either before or during the process.

Data collection

A structured questionnaire was developed (Appendix 1), largely based on one used successfully in a similar study.[1] The questionnaire was designed to capture the number and nature of wounds and the care provided in terms of workforce, service configuration, assessments used (i.e. ankle brachial pressure index (ABPI)), treatment choices, and product usage. Survey forms were delivered to all clinical areas across the five community NHS Trusts. Data were collected for all patients receiving one or more episodes of wound care for their complex wound(s) from an NHS community service during a two-week period. Forms were completed, however, following the consultation during office time; without the patient present and were anonymised at source. To reduce duplication, discussions took place with services providing shared care to minimise the chance of data being collected twice for the same person. This survey was defined as service

For peer review only - http://bmjopen.bmj.com/site/about/guidelines.xhtml

review by the Trusts' Research and Development Departments and did not require approval from a Research Ethics Committee.

The survey consisted of structured questions relating to patient demographics, wound identification by type, number and severity. The form also asked for more detail about each patient's most severe wound. Thus if a patent had multiple complex wounds, more detailed information e.g. on wound treatment was only collected for one wound. For patients with multiple wounds of equal severity, HCPs were asked to focus on the largest wound. Information requested relating to investigations undertaken e.g. Doppler-aided measurement of ABPI or treatments provided e.g. providing pressure relieving equipment, are all specific aspects of care carried out in community settings by the HCPs (and their colleagues) responsible for completing the questionnaire and it would be expected that such clinical procedures would be recorded in the patient's community health record. The questionnaire had been used previously[1] and was piloted locally prior to commencement of the survey by a range of care providers. Minor changes were made in the light of feedback received.

Data Analysis

All data were analysed using simple summary statistics; numbers with percentages for categorical data and mean/median values with range for numerical data. Community point prevalence rates per 10,000 population were produced for each wound type, along with 95% confidence intervals. The denominator used for these calculations was 1 935 683 based on total population figures for the five trusts surveyed taken from Health and Social Care Information Centre 2015 data.

Results

Description of community complex wound population

For peer review only - http://bmjopen.bmj.com/site/about/guidelines.xhtml

Overall we recorded data for 3179 patients with a total of 5632 complex wounds (median number of wounds per patient 1, range 1-24), corresponding to an overall community point prevalence (CPP) of 16.4 per 10,000 (95% confidence interval 15.9 to 17.0). People with complex wounds tended to be elderly (median age 74 years) with at least one comorbidity (median 1.0, range 0-9). Cardiovascular disease was the most frequently reported comorbidity (in 1808 patients; 57%) followed by diabetes (817; 26%) and arthritis (641; 20%). Just under a half of the patients with complex wounds were immobile or walked with difficultly (Table 1) and 494 (31%) of 1613 patients who were reported as being fully mobile were receiving home visits from a HCP. Venous leg ulcers were the most prevalent complex wound type (n=612; CPP 3.2 per 10,000; 95% CI 2.9 to 3.4), followed by diabetic foot ulcers (n=488; CPP 2.5 per 10,000; 95% CI 2.3 to 2.7), traumatic wounds (n=428; CPP 2.2 per 10,000; 95% CI 2.0 to 2.4) and pressure ulcers (n=348; CPP 1.8 per 10,000; 95% CI 1.6 to 2.0).

Table 1. Demographic characteristics of patients with at least one complex wound

Characteristic	
Gender (n=2967)	
Male n (%)	1528 (51)
Female n (%)	1439 (49)
Ethnicity (n=3152)	
White British n (%)	2819 (89)
Other n (%)	336 (11)
Age(n=3120)	
Median (range)	74 (1-107)
**Accommodation (n=3157)**	
Own/rented home n (%)	2728 (86)
Nursing/residential home n (%)	348 (11)
Other n (%)	84 (3)
Number of comorbidities (n=3179)	
Median (range)	1 (0-9)
Continence (n=3029)	
No incontinence n (%)	2487 (82)
Urinary or faecal incontinence or both n (%)	542 (18)
Mobility (n=3141)	
Fully mobile n (%)	1613 (51)
Walks with difficulty n (%)	1091 (35)
Immobile n (%)	437 (14)
**Community point prevalence per 10,000 population for most common wound types n (CPP; 95% CI)**	
Venous leg ulcer	612 (3.2; 2.9 to 3.4)
Diabetic foot ulcer	488 (2.5; 2.3 to 2.7)
Traumatic wound	428 (2.2; 2.0 to 2.4)
Pressure ulcer	348 (1.8; 1.6 to 2.0)

For peer review only - http://bmjopen.bmj.com/site/about/guidelines.xhtml
For peer review only

Use of wound dressings for all complex wounds

Wound dressings are applied to all types of complex wound. Of the 3038 patients for whom data on the 'most severe wound' were provided, 1096 (36%) patients were receiving an antimicrobial primary dressing: 383 (13%) a silver dressing and 713 (23%) patients were receiving other antimicrobial dressings such as iodine or honey. There was marked variation in the use of antimicrobial dressings across community services as shown in Figure 1; ranging from 18% of patients in one area to 69% in another. As noted in Box 1, there is currently insufficient evidence to support the use of antimicrobial dressings to promote wound healing;[12]. It is highly uncertain that antimicrobial dressings are clinically or cost effective and there is certainly no good evidence that they improve wound healing or other outcomes.[12] For VLUs, the use of anti-microbial dressings is specifically not recommended.[35]

The assessment and treatment of leg ulcers

A leg ulcer was reported as being the most severe wound in 25% (n=770) of patients with a most severe wound selected. For these patients a Doppler-aided ABPI measurement is a crucial part of the assessment process to rule out significant peripheral arterial disease and determine treatment choice and access (Box 1).[35] In total, of those patients with one or more leg ulcer, 19% (n=150) did not have an ABPI recorded in community-held notes. The frequency of ABPI recording varied across services from 10% to 28% and for a further 21% (n=167) this information was either unknown or not reported (ranging from 13% to 22%).

A VLU was reported as being the most severe wound in 570 patients of whom 175 (31%) were recorded as receiving no compression therapy; ranging from 2% to 30% (compression is an effective first-line treatment for venous ulcers[37 38]). Half (n=287; 50%) of those with VLUs were managed with compression bandages, 79 (14%) with compression hosiery and 29 (5%) with a combination of the two (Figure 2). There was limited use of two layer compression hosiery across all areas surveyed despite its known cost-effectiveness relative to compression bandaging.[33] None of the patients with a diagnosis of VLU were prescribed pentoxifylline; a treatment shown to be clinically and cost effective and recommended to promote the healing of VLUs.[35]

For peer review only - http://bmjopen.bmj.com/site/about/guidelines.xhtml

Use of support surfaces in those at risk of pressure ulceration

Over one third (35%) of patients with complex wounds were reported as being at risk of pressure ulceration and 348 (11%) had a pressure ulcer at the time of survey. Of the 281 patients whose most severe wound was a pressure ulcer (who are thus at known high risk of further ulceration and should be receiving pressure relief[22]), 109 (39%) patients were reported as not having a pressure-relieving cushion or mattress (ranging from 27% in one area to 64% in another; Figure 3). For the 711 patients with a foot ulcer selected as the most severe wound, 40% (n=286) were not receiving any pressure relief for the affected foot (ranging from 31% to 60%).

Discussion

This study characterised the number and nature of complex wounds being cared for by NHS community services, and the assessments and treatments being used in their management. Our estimate of the point prevalence of complex wounds (16.4 cases per 10,000 population) is consistent with another estimate from the North of England (14.7 per 10.000 population[1]). As these are point prevalence estimates from general populations using census figures as denominators, the estimates are lower than those estimates which focus on specific high-risk populations (such as hospital patients).[39] We found indicators that revealed unwarranted variation in clinical practice across participating services, the underuse of evidence-based interventions (e.g., compression therapy for venous ulcers; documentation of ABPI) and the overuse of interventions supported by limited evidence (e.g., antimicrobial dressings).

Overuse of interventions supported by limited evidence

Across community services, the survey revealed substantial variation in the use of antimicrobial dressings for wound management, ranging from 18% to 69% of all primary dressings used. Antimicrobial dressings often cost much more than their non-antimicrobial counter parts, however, their use is associated with a high cost for little or no known patient benefit. To illustrate the type of impact of this use we considered silver

For peer review only - http://bmjopen.bmj.com/site/about/guidelines.xhtml

dressings for which current evidence on the relative effectiveness for preventing or treating wound infection and promoting healing is uncertain.[40 41] Silver dressings cost several times more than their non-silver counterparts. In a scenario where silver dressings are used for a patient over a 6-week period with three dressing changes per week, the dressing cost (using an average cost for a small dressing) would be approximately £146. The cost for a standard foam dressing (of the same size) in the same scenario is approximately £31. Use of silver dressings in this situation (assuming all other aspects of care and outcome are equal) corresponds to an additional £11,500 per 100 patients treated with silver dressings. Tackling overuse is not an easy task and not solely the responsibility of individual HCPs. But despite increased awareness amoung clinicians, policy makers and the general public during the last 5-10 years there have not been widespread changes in policy to address this issue.[42] In wound care specifically, removing silver dressings from a prescribing formulary would offer a partial solution only. There is a clear need for a rational strategy for identifying, assessing and disinvesting from products supported by little or no evidence; ideally this should be delivered at a health system level.[43]

Underuse of evidence-based interventions

Across our population we saw underuse of ABPI in those with leg ulcers (with 19% not having this measure recorded and for 21% it was unclear whether a recording had been taken). Such under-assessment (whether a delay or omission) is likely linked to the underuse of compression therapy also observed. These related issues require urgent attention since compression is a highly efficient and cost-effective treatment for VLU.[35] The first response to this finding is to seek an understanding of the reasoning behind the observed patterns of compression use. For example, underuse may be due to patients waiting for ABPI assessment, or ABPI assessment identifying peripheral arterial disease that may preclude compression use; in other cases, lack of compression may reflect issues with patient adherence to therapy or a shortage of staff with compression bandaging skills. The extent to which current findings can be explained by appropriate contraindications is unclear and requires further investigation. Crucially the reason for such wide variation between relatively local areas also needs further explanation.

For peer review only

Using data from randomised controlled trials,[44 45] we conservatively calculate that non-use of compression in those with VLUs will lead to 23 fewer people per 100 patients with VLU healing over 12 months compared with use of compression. Using an estimated mean cost per VLU episode of £1800[33] we can estimate an annual increased cost of £41,400 per 100 VLU patients not using compression. However, we could tentatively also consider non-use of compression as a proxy for sub-optimal care. If we apply the recent Rightcare[26] estimated cost savings associated with optimised VLU care (including high compression use), savings could be as high as £118,979 per 100 VLU patients. While here we have focused on cost impacts, it is important to consider the improved health-related quality of life associated with a healed wound as well as the reduced financial burden on patients, especially those who are unable to work or require time away from work to attend clinic appointments.

There is also an apparent underuse of pentoxifylline, a xanthine derivative used to treat muscle pain in people with peripheral arterial disease, but also believed to increase microcirculatory blood flow in patients with VLU. Uptake is low despite evidence from 12 RCTs included in a systematic review[46] that it increases ulcer healing both with and without compression. The reasons for this underuse are being explored further but are likely to include: lack of awareness of the drug and its effects amongst both community GPs and nurses; relative lack of nurse prescribers able to prescribe pentoxifylline and reluctance to prescribe for an off-licence indication (particularly in people who may be taking several other medicines for comorbidities). It is also worth noting that the drug is low cost but not marketed because the patent is long expired. It is unclear whether this treatment is used more widely outside the UK. Using published relative effectiveness estimates and related costs,[47] we have estimated that use of pentoxifylline alongside compression could result in cost savings of over £40,000 per 100 VLU patients treated. Combined increases in the use of ABPI, cost-effective compression therapy and greater pentoxifylline use in the treatment of those with venous leg ulceration is likely to see a cumulative increase in improved patient outcomes, experiences and reduced health care costs.

For peer review only - http://bmjopen.bmj.com/site/about/guidelines.xhtml

Similar exploration is required in relation to the potential underuse of pressure relieving equipment suggested by our study, in order to understand the true scope for improvement. In all cases, further work is required to identify the factors that underpin clinical decision making and behaviour in these areas. An understanding of such factors would support the identification and selection of appropriate behaviour change techniques and implementation strategies, targeted at modifying these behaviours and generating improvement and thus value in healthcare. Having a pressure ulcer is prognostic for further pressure ulceration, thus those with an ulcer are considered at risk and should receive pressure-relieving interventions such as support surfaces. Even accounting for the initial cost of purchasing a device given the large cost associated with healing a pressure ulcer (estimated cost for healing a Grade 2 pressure ulcer = £5,241),[48] we conservatively calculate that the use of support surfaces to prevent pressure ulceration could save at least £38,000 per 100 at-risk patients. Again those with a pressure ulcer are also known to have worse health-related quality of life than others with similar co-morbidities.[49] These are not isolated examples of underuse, a US study found that patients with acute or chronic conditions (that represented the leading causes of illness, death and utilization of healthcare) received only 55% of recommended care[50] and similarly the CareTrack study found that Australians received appropriate care in only 57% of 35,573 eligible healthcare encounters.[51] However, this is the first work that has revealed so clearly, variation in the use of interventions for VLUs where there are corresponding guideline-based recommendations. Failure to deliver best practice is often a result of poor execution or lack of widespread adoption of best care processes.[52] International interest in research translation and quality improvement reflects the growing recognition of the slow and inconsistent uptake of effective healthcare practices worldwide.[53]

Disinvestment and implementation of improvement initiatives to promote better value care

Local variation in product choice between organisations is shown clearly in the work presented. This highlights the huge scope for better value care in both the assessment and treatments of complex wounds; value could be released by disinvestment in some areas with savings being focused on areas of underuse identified here. Further work should be undertaken to understand the factors that underpin decision making

For peer review only - http://bmjopen.bmj.com/site/about/guidelines.xhtml

around treatment use, with a particular focus on exploring the motivations to use expensive treatments with limited evidence at a time when the NHS faces significant resource constraints. Working with our service partners, we plan to address the observed local practice variations through a programme of improvement work undertaken as part of NIHR CLAHRC GM. However, we recognise that this process needs to be replicated across the health system as a whole if overuse and underuse are to be addressed fully. As we have stated above, there is a clear need for a national strategy for identifying, assessing and disinvesting from products and practices supported by little or no evidence.[43] A model for taking a systematic approach to disinvestment already exists in the form of the SHARE programme in Australia, This model enables clinicians, managers and policy makers to manage the process from identifying the need for disinvestment to implementing the change and evaluating outcomes.[54] Similar efforts are required in the UK if practice variations are to be addressed

Strengths and limitations

This cross-sectional survey provides robust community-focused population point-prevalence estimates for different types of complex wounds and is the first multi-service survey to capture the wide variation in treatment and care of complex wounds between different NHS Trusts. There are a few limitations to our study. Firstly, the study looked specifically at patients receiving care from NHS community services and did not include people whose wound care was delivered by hospitals, primary care, or other care providers, and also those who may have been self-treating at this time. While this prevents us from comparing the characteristics of people with and without complex wounds at the time of the survey, collection of these data would have been too resource intensive for participating services and we know from previous work that the vast majority of people with wounds are in community settings.[39] Secondly, we only scrutinised the patient information readily available to community staff and did not examine wider (e.g., GP) patient records. However given that we were looking for information regarded as crucial to the management of people with complex wounds, any omission of information from community records risks suboptimal clinical decision making. Thirdly, it is also conceivable that services failed to complete a survey form for each patient they saw with a complex wound during the survey period. However, both the level of engagement from

For peer review only - http://bmjopen.bmj.com/site/about/guidelines.xhtml

services and the similarity between the estimated CPP obtained here and that found in a previous but smaller survey in Leeds,[1] suggest the impact of this is likely to be negligible. Finally, as this was an anonymised survey we were unable to neither conduct any case validation nor validate wound aetiology. Consequently, data presented reflects the treating HCP's assessment.

Conclusions

This survey adds important robust epidemiological data to the complex wound literature where existing prevalence data have been found to be limited when systematically reviewed.[39] We also highlight the over- and underuse of services and treatments and reflect the issues emphasised in a number of reports relating to Better Value Healthcare.[17 24 27] These findings suggest significant opportunities for delivering better value wound care exist. Efforts should now focus on developing strategies to identify, assess and disinvest from products and practices supported by little or no evidence and enhance the uptake of those that are.

Figure Legends

Figure 1. Proportion of complex wounds for which primary dressing contained antimicrobials (bars represent included community services), *Number of patients per community service ranged from 172 to 655*

Figure 2. Highest level of compression used for patients with venous leg ulcers selected as most severe wound (bars represent included community services). *Number of patients per community service ranged from 14 to 151*

Figure 3. Proportion of patients using pressure relieving mattresses or cushions (bars represent included community services). *Number of patients per community service ranged from 10 to 63*

References

1. Hall J, Buckley HL, Lamb KA, et al. Point prevalence of complex wounds in a defined United Kingdom population. *Wound Repair Regen* 2014;22(6):694-700. doi: 10.1111/wrr.12230 [published Online First: 2014/09/17]
2. Guest JF, Ayoub N, McIlwraith T, et al. Health economic burden that wounds impose on the National Health Service in the UK. *BMJ Open* 2015;5(12):e009283. doi: 10.1136/bmjopen-2015-009283
3. Graves N, Zheng H. Modelling the direct health care costs of chronic wounds in Australia. *Wound Practice and Research* 2014; 22(1).

For peer review only - http://bmjopen.bmj.com/site/about/guidelines.xhtml

4. Sen CK, Gordillo GM, Roy S, et al. Human skin wounds: a major and snowballing threat to public health and the economy. *Wound Repair Regen* 2009;17(6):763-71. doi: 10.1111/j.1524-475X.2009.00543.x

5. WHO. Global Health and Aging: National Institute on Aging, National Institutes of Heath, US Department of Health and Human Services, World Health Organisation, 2011.

6. Gould L, Abadir P, Brem H, et al. Chronic wound repair and healing in older adults: current status and future research. *J Am Geriatr Soc* 2015;63(3):427-38. doi: 10.1111/jgs.13332

7. Corbett LQ. Wound Care Nursing: Professional Issues and Opportunities. *Advances in Wound Care* 2011;1(5):189-93.

8. Harrison MB, Graham ID, Lorimer K, et al. Nurse clinic versus home delivery of evidence-based community leg ulcer care: a randomized health services trial. *BMC Health Serv Res* 2008;8:243. doi: 10.1186/1472-6963-8-243

9. Medical Advisory Secretariat. Community-based care for chronic wound management: an evidence-based analysis. *Ontario Health Technology Assessment Series* 2009;9(18)

10. Moore Z, Butcher G, Corbett LQ, et al. AAWC, AWMA, EWMA Position Paper: Managing Wounds as a Team *J Wound Care* 2014;23:S1-S38.

11. Whitlock E, Morcom J, Spurling G, et al. Wound care costs in general practice - a cross-sectional study. *Aust Fam Physician* 2014;43(3):143-6.

12. NICE. Chronic wounds: advanced wound dressings and antimicrobial dressings: Evidence summary [ESMPB2]: National Institute for Health and Care Excellence, 2016.

13. Erstad BL. Value-Based Medicine: Dollars and Sense. *Crit Care Med* 2016;44(2):375-80. doi: 10.1097/CCM.0000000000001559

14. Gabbay J, le May A. Evidence based guidelines or collectively constructed "mindlines?" Ethnographic study of knowledge management in primary care. *BMJ* 2004;329(7473):1013. doi: 10.1136/bmj.329.7473.1013

15. Thompson C, Cullum N, McCaughan D, et al. Nurses, information use, and clinical decision making--the real world potential for evidence-based decisions in nursing. *Evid Based Nurs* 2004;7(3):68-72.

16. Enthoven AC. Integrated delivery systems: the cure for fragmentation. *Am J Manag Care* 2009;15(10 Suppl):S284-90.

17. Porter ME. What is value in health care? *N Engl J Med* 2010;363(26):2477-81. doi: 10.1056/NEJMp1011024

18. Prasad V, Vandross A, Toomey C, et al. A decade of reversal: an analysis of 146 contradicted medical practices. *Mayo Clin Proc* 2013;88(8):790-8. doi: 10.1016/j.mayocp.2013.05.012

19. Productivity Commission. Impacts of Advances in Medical Technology in Australia. Research Report 2005 [Available from: http://www.pc.gov.au/inquiries/completed/medical-technology. (accessed May 2017) accessed May 2017 2017.

20. WHO. Appropriateness in Health Care Services. In: WHO Regional Office for Europe, ed. Copenhagen, Denmark World Health Organisation, 2000.

21. Muir Gray JA. How to Get Better Value Healthcare Second ed. Oxford, UK: Offox Press for Better Value Healthcare Ltd 2011.

22. NICE. Pressure ulcers: prevention and management Clinical guideline [CG179]: National Institute for Health and Care Excellence, 2014.

23. Ubbink DT, Santema TB, Stoekenbroek RM. Systemic wound care: a meta-review of cochrane systematic reviews. *Surg Technol Int* 2014;24:99-111.

24. Alderwick H, Robertson R, Appleby J, et al. Better value in the NHS: The role of changes in clinical practice. London, UK: The King's Fund, 2015.

25. Cripps M. NHS Right Care – expanding the approach in the context of delivering the Five Year Forward View: NHS England; 2015 [Available from: https://www.england.nhs.uk/wp-content/uploads/2015/12/nqb-oct15-5.pdf (accessed April 2017) accessed 24th April 2017.

26. NHS RightCare. NHS RightCare scenario: The variation between sub-optimal and optimal pathways. Betty's story: Leg ulcer wound care NHS England; 2017 [Available from: https://www.england.nhs.uk/rightcare/wp-content/uploads/sites/40/2017/01/nhs-rightcare-bettys-story-narrative-full.pdf (accessed May 2017).

27. Levinson W, Kallewaard M, Bhatia RS, et al. 'Choosing Wisely': a growing international campaign. *BMJ Qual Saf* 2015;24(2):167-74. doi: 10.1136/bmjqs-2014-003821

28. ABIM Foundation. Choosing Wisely Philadelphia: ; 2017 [Available from: http://www.choosingwisely.org/ (accessed June 2017) accessed 18th May 2017 2017.

29. Canadian Medical Association. Choosing Wisley Canada University of Toronto 2017 [Available from: http://www.choosingwiselycanada.org/ (accessed May 2017).

30. Bhatia RS, Levinson W, Shortt S, et al. Measuring the effect of Choosing Wisely: an integrated framework to assess campaign impact on low-value care. *BMJ Qual Saf* 2015;24(8):523-31. doi: 10.1136/bmjqs-2015-004070

31. Hodgson R, Allen R, Broderick E, et al. Funding source and the quality of reports of chronic wounds trials: 2004 to 2011. *Trials* 2014;15:19. doi: 10.1186/1745-6215-15-19

32. Lazarus GS, Kirsner RS, Zenilman J, et al. Clinical interventions for venous leg ulcers: Proposals to improve the quality of clinical leg ulcer research. *Wound Repair Regen* 2016;24(5):767-74. doi: 10.1111/wrr.12466

For peer review only - http://bmjopen.bmj.com/site/about/guidelines.xhtml

33. Ashby RL, Gabe R, Ali S, et al. Clinical and cost-effectiveness of compression hosiery versus compression bandages in treatment of venous leg ulcers (Venous leg Ulcer Study IV, VenUS IV): a randomised controlled trial. *Lancet* 2014;383(9920):871-9. doi: 10.1016/S0140-6736(13)62368-5
34. NICE. Diabetic foot problems: prevention and management [NG19]: National Institute for Health and Care Excellence, 2015.
35. SIGN. Management of chronic venous leg ulcers: A national clinical quideline (120): Scottish Intercollegiate Guidelines Network, 2010.
36. Kelly J, Clifton E, Fearns N, Harbour J, Heller-Murphy S, Herbert P, Macpherson K, Myles S, Santo V, Wilson L Antimicrobial wound dressings (AWDs) for chronic wounds. Health Technology Assessment report 13: Health Improvement Scotland, 2015.
37. Nelson EA, Bell-Syer SE. Compression for preventing recurrence of venous ulcers. *Cochrane Database Syst Rev* 2014(9):CD002303. doi: 10.1002/14651858.CD002303.pub3
38. O'Meara S, Cullum N, Nelson EA, et al. Compression for venous leg ulcers. *Cochrane Database Syst Rev* 2012;11:CD000265. doi: 10.1002/14651858.CD000265.pub3
39. Cullum N, Buckley H, Dumville J, et al. Wounds Research for Patient Benefit: a 5 year programme of research. *Programme Grants Appl Res* 2016;4(13)
40. Storm-Versloot MN, Vos CG, Ubbink DT, et al. Topical silver for preventing wound infection. *Cochrane Database Syst Rev* 2010(3):CD006478. doi: 10.1002/14651858.CD006478.pub2
41. Vermeulen H, van Hattem JM, Storm-Versloot MN, et al. Topical silver for treating infected wounds. *Cochrane Database Syst Rev* 2007(1):CD005486. doi: 10.1002/14651858.CD005486.pub2
42. Brownlee S, Chalkidou K, Doust J, et al. Evidence for overuse of medical services around the world. *Lancet* 2017 doi: 10.1016/S0140-6736(16)32585-5
43. Prasad V, Ioannidis JP. Evidence-based de-implementation for contradicted, unproven, and aspiring healthcare practices. *Implement Sci* 2014;9:1. doi: 10.1186/1748-5908-9-1
44. Ashby RL, Gabe R, Ali S, et al. VenUS IV (Venous leg Ulcer Study IV) - compression hosiery compared with compression bandaging in the treatment of venous leg ulcers: a randomised controlled trial, mixed-treatment comparison and decision-analytic model. *Health Technol Assess* 2014;18(57):1-293, v-vi. doi: 10.3310/hta18570
45. Morrell CJ, King B, Brereton L. Community-based leg ulcer clinics: organisation and cost-effectiveness. *Nurs Times* 1998;94(9):51-4.
46. Jull AB, Arroll B, Parag V, et al. Pentoxifylline for treating venous leg ulcers. *Cochrane Database Syst Rev* 2012;12:CD001733. doi: 10.1002/14651858.CD001733.pub3
47. Iglesias CP, Birks Y, Nelson EA, et al. Quality of life of people with venous leg ulcers: a comparison of the discriminative and responsive characteristics of two generic and a disease specific instruments. *Qual Life Res* 2005;14(7):1705-18.
48. Bennett G, Dealey C, Posnett J. The cost of pressure ulcers in the UK. *Age Ageing* 2004;33(3):230-5. doi: 10.1093/ageing/afh086
49. Essex HN, Clark M, Sims J, et al. Health-related quality of life in hospital inpatients with pressure ulceration: assessment using generic health-related quality of life measures. *Wound Repair Regen* 2009;17(6):797-805. doi: 10.1111/j.1524-475X.2009.00544.x
50. McGlynn EA, Asch SM, Adams J, et al. The quality of health care delivered to adults in the United States. *N Engl J Med* 2003;348(26):2635-45. doi: 10.1056/NEJMsa022615
51. Runciman WB, Hunt TD, Hannaford NA, et al. CareTrack: assessing the appropriateness of health care delivery in Australia. *Med J Aust* 2012;197(2):100-5.
52. Berwick DM, Hackbarth AD. Eliminating waste in US health care. *JAMA* 2012;307(14):1513-6. doi: 10.1001/jama.2012.362
53. Glasziou P, Straus S, Brownlee S, et al. Evidence for underuse of effective medical services around the world. *Lancet* 2017 doi: 10.1016/S0140-6736(16)30946-1
54. Harris C, Green S, Ramsey W, et al. Sustainability in Health care by allocating resources effectively (SHARE) 1: introducing a series of papers reporting an investigation of disinvestment in a local healthcare setting. *BMC Health Serv Res* 2017;17(1):323. doi: 10.1186/s12913-017-2210-7

For peer review only

For peer review only - http://bmjopen.bmj.com/site/about/guidelines.xhtml

Funding

This project was funded by the National Institute for Health Research Collaboration for Leadership in Applied Health Research and Care (NIHR CLAHRC) Greater Manchester. The NIHR CLAHRC Greater Manchester is a partnership between providers and commissioners from the NHS, industry and the third sector, as well as clinical and research staff from the University of Manchester. The views expressed in this article are those of the author(s) and not necessarily those of the NHS, NIHR or the Department of Health.

Contributors

NC and JD conceived the idea and design for the overall project, TG, KR and PW contributed to further development of the study design, TG and KR conducted stakeholder consultation with practitioners, SR was responsible for data analysis, RA and JD contributed to data analysis. TG created the original draft of the manuscript. All authors contributed to the interpretation of study findings, critical revision of the manuscript for important intellectual content and approval of the final manuscript.

Acknowledgements

The authors are grateful to the healthcare professionals, from NHS partner organisations, involved in data collection, for giving their time and enthusiastically supporting this project. The authors would also like to thank Wanda Russell (NIHR CLAHRC GM Research Fellow at the time of this project) for her role in conducting stakeholder consultation with practitioners and data collection and Louise Hussey, Research Fellow, for her contribution to data analysis.

Competing interests

None declared.

For peer review only

Data sharing statement

Requests for access to data should be addressed to the corresponding author.

For peer review only - http://bmjopen.bmj.com/site/about/guidelines.xhtml

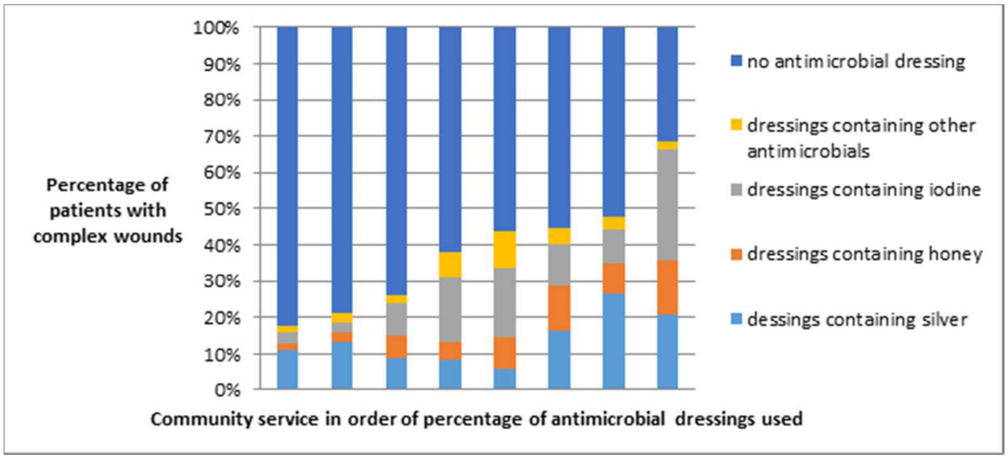

52x23mm (300 x 300 DPI)

For peer review only

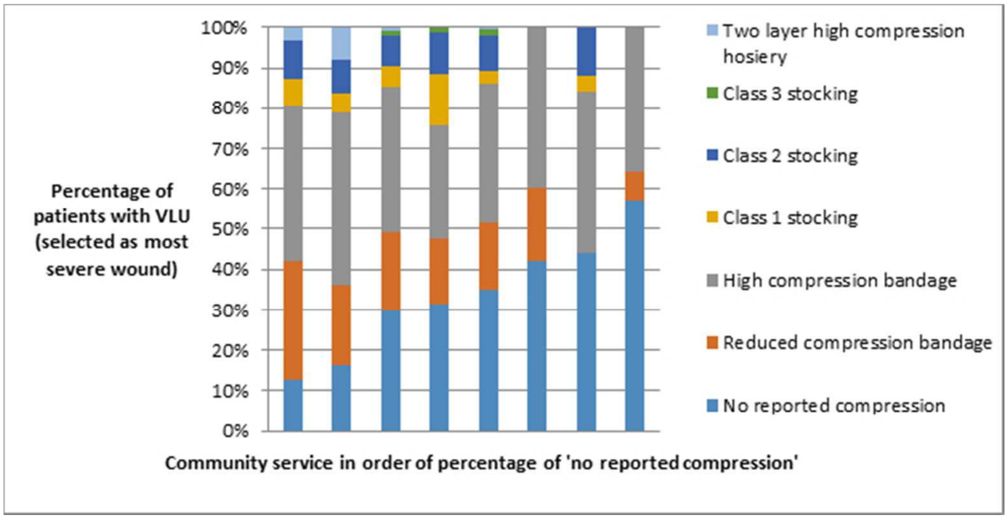

52x27mm (300 x 300 DPI)

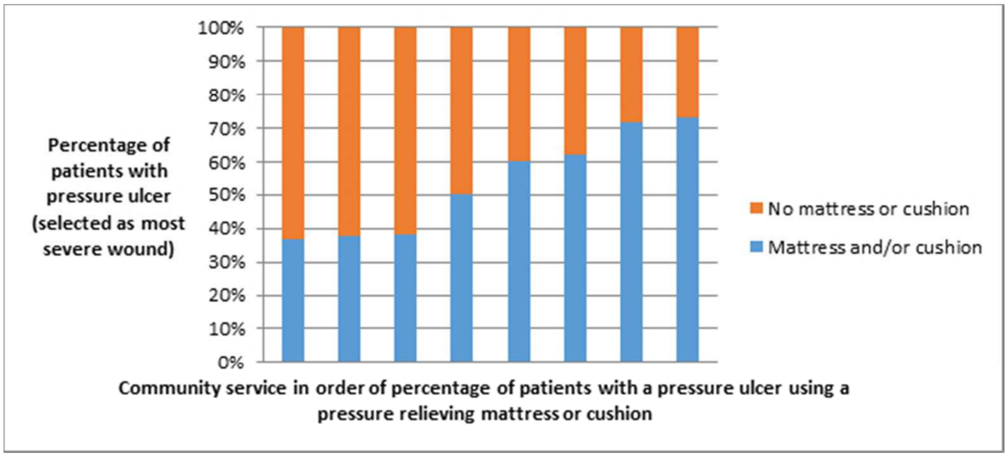

52x23mm (300 x 300 DPI)

For peer review only

Office use only: ID number

☐☐☐☐

The University of Manchester

NHS National Institute for Health Research
Collaboration for Leadership in Applied Health Research and Care (CLAHRC) Greater Manchester

A Survey of Complex Wounds and their Care in the Community

Patient Data Capture Form

Date this form was completed: ☐☐ / ☐☐ / ☐☐☐☐
day *month* *year*

This form is to be completed by the NHS health care professional (from the participating NHS community-based services) delivering most of the hands on wound care for a person with one or more complex wounds. <u>Please aim to ensure that only one form is completed per patient</u>

Section 1:

1.1 What is your main clinical role currently? *(Please cross (x) one option)*

District Nurse/community nurse ☐ Nurse Specialist ☐

Podiatrist ☐ Other ☐

If 'Other' please state: ☐

1.2 What is your NHS Agenda for Change band (as the health care professional from the participating NHS community-based services delivering most of the hands on wound care for this patient)? *(Please cross (x) one option)*

Band 3 ☐ Band 4 ☐ Band 5 ☐ Band 6 ☐ Band 7 ☐ Band 8a ☐

Other ☐ If 'Other' please state: ☐

1.3 Currently, in which location does this patient receive <u>most</u> of their hands on wound care? *(Please cross (x) one option)*

Patient's home ☐ Community clinic/heath centre/GP practice ☐ Nursing home ☐

Residential home ☐ Hospice ☐ Prison ☐

Other ☐ If 'Other' please state: ☐

For peer review only - http://bmjopen.bmj.com/site/about/guidelines.xhtml

1.4 How many wound related consultations (with all professionals involved) has THIS patient had in the last seven days?: ☐☐

1.5 Duration of the most recent consultation: ☐☐☐ minutes

1.6 Are any other health professionals involved in deciding or planning wound care for this person? *(Please cross (x) one option)*

Yes ☐ **No** ☐ Don't know ☐

↓

1.7 If Yes who are they? *(please cross (x) all that apply)*:

GP ☐ Tissue viability nurse ☐ Other wound care specialist ☐

Podiatrist ☐ District nurse ☐

Other ☐ If 'Other' please state: ☐

Section 2: Patient details

2.1 Patient's DOB: dd ☐☐ mm ☐☐ yyyy ☐☐☐☐ **2.2 Patient's gender:** male ☐ female ☐

2.3 Ethnicity:

White British ☐	White Irish ☐	White Other ☐	Black African ☐	Black Caribbean ☐	Black Other ☐
Asian Indian ☐	Asian Pakistani ☐	Asian Bangladeshi ☐	Asian Other ☐	White and Black Caribbean ☐	White and Black African ☐
White and Asian ☐	Other mixed background ☐	Chinese ☐	Other ☐		

2.4 Please state location of patient's GP *(Please cross (x) one option)*

Central Manchester ☐ South Manchester ☐ Salford ☐ Oldham ☐

Bury ☐ Heywood, Middleton, Rochdale ☐ Trafford ☐

Other location ☐ If other, please state: ☐

Don't know ☐

For peer review only - http://bmjopen.bmj.com/site/about/guidelines.xhtml

For peer review only

2.5 What type of accommodation best describes where this patient currently lives? *(Please cross (x) one option)*

Own / rented home ☐ Nursing home ☐ Residential home ☐

No fixed abode ☐ Prison ☐

Other ☐ If 'Other' please state: ☐

2.6 What co-morbidities does this patient have? *(please cross (x) all that apply)*:

**Cardiovascular disease (CVD)**	e.g. Hypertension (high blood pressure, high BP), Myocardial infarction (MI, heart attack, IHD), Angina (IHD), Heart failure (CCF, CHF, HF, LVF)	☐
**Peripheral Arterial Disease**	e.g. Ischaemia (leg/foot), Claudication (intermittent claudication), Rest pain, Arteriopathy/angiopathy, Aneurysm including aortic aneurysm	☐
**Diabetes (DM)**	e.g. Insulin dependent DM (IDDM, Type I diabetes), Non insulin dependent DM (NIDDM, Type II diabetes)	☐
**Arthritis**	e.g. Rheumatoid arthritis (RA), Osteoarthritis (OA), Non rheumatoid arthritis	☐
**Orthopaedics**	e.g. Fractured neck of femur (# NOF, # hip, fractured hip, broken hip), Other fracture/break, Elective orthopaedic	☐
**Airways**	e.g. Chronic obstructive airways/pulmonary disease (COAD, COPD), Chronic bronchitis, Emphysema, Asthma	☐
**Neurological**	e.g. Parkinson's disease, Multiple sclerosis (MS), Epilepsy (fits, Grand mal, Petit mal, tonic-clonic, seizure), Spinal Injury	☐
**Stroke**	e.g. (cerebrovascular accident, CVA, transient ischaemic attack, TIA)	☐
**Cancer**	e.g. any cancer diagnosis	☐

For peer review only

2.7 Patient's Continence *(please cross (x) all that apply)*:

No incontinence ☐ Urinary incontinence ☐ Faecal Incontinence ☐

2.8 Patient mobility *(please cross (x) one option)*:

Patient walks freely ☐ Patient walks with difficultly ☐ Patient is immobile ☐

2.9 In your judgement is this patient at risk of pressure ulceration? *(please cross (x) one option)*:

Yes ☐ No ☐ Don't know ☐

↓

2.10 If yes is their pressure ulcer risk documented in their notes/care plan? *(please cross (x) one option)*:

Yes ☐ No ☐ Don't know ☐

Section 3: Current complex wounds

3.1 Please record the number of wounds (by wound type) that this patient has.

Wound type:		Number of wound of this type (please circle one number for each type or leave blank if 0):							
Foot ulcer in patient with diabetes	RIGHT foot	1	2	3	4	5	6	7	8 or more
	LEFT foot	1	2	3	4	5	6	7	8 or more
Foot ulcer in patient without diabetes	RIGHT foot	1	2	3	4	5	6	7	8 or more
	LEFT foot	1	2	3	4	5	6	7	8 or more
Venous leg ulcer	RIGHT leg	1	2	3	4	5	6	7	8 or more
	LEFT leg	1	2	3	4	5	6	7	8 or more
Arterial leg ulcer	RIGHT leg	1	2	3	4	5	6	7	8 or more
	LEFT leg	1	2	3	4	5	6	7	8 or more
Arterial/venous (mixed aetiology) leg ulcer	RIGHT leg	1	2	3	4	5	6	7	8 or more
	LEFT leg	1	2	3	4	5	6	7	8 or more
Pressure ulcer		1	2	3	4	5	6	7	8 or more

For peer review only

**Dehisced surgical wound currently healing by secondary intention**	1	2	3	4	5	6	7	8 or more
**Wound resulting from excision of pilonidal sinus**	1	2	3	4	5	6	7	8 or more
**Wound resulting from excision of peri-anal abscess**	1	2	3	4	5	6	7	8 or more
**Other surgical wound healing by secondary intention**	1	2	3	4	5	6	7	8 or more
**Traumatic wound (e.g. result of road traffic accident)**	1	2	3	4	5	6	7	8 or more
**Fungating carcinoma**	1	2	3	4	5	6	7	8 or more
**Burn**	1	2	3	4	5	6	7	8 or more

**Other wound type - please describe:**	**Number of wound of this type (please circle):**							
	1	2	3	4	5	6	7	8 or more
	1	2	3	4	5	6	7	8 or more
	1	2	3	4	5	6	7	8 or more

For peer review only

Section 4: Treatment of complex wounds

We now want to collect information on the treatment of this patient's complex wound(s). If the patient has more than one complex wound (of any type) please now focus on the one wound you consider to be the worst/most severe (if there are more than one wound of equal severity choose any one of these wounds and complete the following information for just this wound).

We will refer to the wound for which the following data are collected as the selected wound.

4.1 Please record what type of wound the selected wound is (as noted above - this is the most severe/wound and therefore the one you are focusing on in this section). ***Please cross (x) one option.***

Foot ulcer in patient with diabetes	☐	Foot ulcer in patient without diabetes	☐
Venous leg ulcer	☐	Arterial leg ulcer	☐
Arterial/venous leg ulcer	☐	Pressure ulcer	☐
Dehisced surgical wound currently healing by secondary intention	☐	Wound resulting from excision of pilonidal sinus	☐
Wound resulting from excision of peri-anal abscess	☐	Other surgical wound healing by secondary intention	☐
Traumatic wound (e.g. result of road traffic accident)	☐	Fungating carcinoma	☐
Burn	☐		
Other wound type	☐	If 'Other' please state:	

4.2 Please note the location/area most affected by the selected wound. ***Please cross (x) one option.***

head/neck	☐	arm	☐	hand	☐	breast	☐
abdomen	☐	back	☐	sacrum	☐	natal cleft	☐
buttock	☐	peri-anal	☐	perineum	☐	leg	☐
foot	☐	Other	☐	If 'Other' please state:			

Please record the following details for the selected wound. ***Please cross (x) one option.***

4.3 Exudate level:

High	☐	Moderate/ medium	☐	Low	☐	None	☐

4.4 Malodour:

Yes	☐	No	☐

For peer review only

4.5 For the selected wound, which wound dressings are being used currently? ***(Refer to the list of dressings in the guidelines and insert the corresponding code in each of the boxes)*:**

Primary dressing ☐☐ Secondary dressing ☐☐

Please record any other wound treatments for the selected wound:

4.6 Drugs/medicines: ***(please cross (x) all that apply)***

Antibiotics for wound infections ☐ Pentoxifylline ☐

Topical steroids ☐ Analgesics ☐

Other ☐ If 'Other' please state: ☐

4.7 For the selected wound, the bandage currently used: ***(Please cross (x) one option).***

N/A ☐ 4 layer compression bandage ☐

Short stretch bandage (e.g. Actico/Comprilan) ☐ 3 layer reduced compression bandage ☐

2 layer compression bandage (e.g. K2) ☐ Non-compression bandage (plus wadding) ☐

Dressing retention bandage (e.g. K-band) ☐

Other ☐ If 'Other' please state: ☐

4.8 Hosiery (stockings) for the leg affected by the selected wound: ***(Please cross (x) one option).***

N/A ☐ Standard class 1 ☐

Standard class 2 ☐ Standard class 3 ☐

Made to measure Class 1 ☐ Made to measure Class 2 ☐

Made to measure Class 3 ☐ Standard two layer compression hosiery (aiming to deliver 40mmhg) ☐

Made to measure two layer compression hosiery (aiming to deliver 40mmhg) ☐

Other ☐ If 'Other' please state: ☐

For peer review only - http://bmjopen.bmj.com/site/about/guidelines.xhtml

Please record any equipment used relating to the selected wound:

4.9 Pressure relief and mobility: ***(please cross (x) all that apply)***

Pressure relieving	**mattresses:**	**cushions:**
High specification foam replacement e.g. MSS gilde	☐	☐
Air alternating replacement e.g. Quattro deep cell prime	☐	☐
Air alternating overlay e.g. Alpha excel	☐	☐
Repose (overlay system)	☐	☐
Foam overlay	☐	☐

Crutches ☐

Wheelchair ☐

Other ☐

If 'Other' please state: ______

4.10 Footwear/Orthotics/Foot pressure reliving equipment: ***(please cross (x) all that apply)***

Prescribed footwear ☐

Footwear adaptation ☐

Insoles ☐

Heel pressure relief ☐ *e.g. Repose Heel Trough, PROFO boot*

Other ☐ If 'Other' please state: ______

4.11 Other therapies for the selected wound: ***(please cross (x) all that apply)***

Hyperbaric oxygen therapy ☐

Physiotherapy ☐

Occupational therapy ☐

Negative pressure wound therapy ☐

Other ☐ If 'Other' please state: ______

For peer review only

4.12 Treatment objectives for the selected wound: ***(please cross (x) all that apply)***

Debridement ☐ Absorption (removal of exudate) ☐

Hydration (keep the wound moist) ☐ Microbial load management ☐

Odour Management ☐ Reduce overgranulation ☐

Encourage granulation ☐ Protection ☐

Other ☐ If 'other' please state: ☐

For peer review only

4.13 If the selected wound is a PRESSURE ULCER record its category here (otherwise leave blank). (*Please cross (x) one option).*

I ☐ II ☐ III ☐ IV ☐ Unstageable ☐ Not applicable ☐

If the selected wound is a LEG ULCER please record the following (otherwise leave blank):

4.14 Is a Doppler ABPI for the leg with the selected wound recorded in the notes? (*Please cross (x) one option).*

Yes ☐ No ☐ Don't know ☐

4.15 If yes – please note the ABPI measure here:

☐☐ • ☐☐

BMJ Open

OPPORTUNITIES FOR BETTER VALUE WOUND CARE: a multi-service, cross sectional survey of complex wounds and their care in a UK community population

Journal:	*BMJ Open*
Manuscript ID	bmjopen-2017-019440.R1
Article Type:	Research
Date Submitted by the Author:	12-Dec-2017

Complete List of Authors:	Gray, Trish; University of Manchester, Division of Nursing, Midwifery and Social Work, School of Health Sciences, Faculty of Biology, Medicine and Health; NIHR CLAHRC Greater Manchester<br>Rhodes, Sarah; University of Manchester, Centre for Biostatistics, Faculty of Biology, Medicine and Health; NIHR CLAHRC Greater Manchester<br>Atkinson, Ross; University of Manchester, Division of Nursing, Midwifery and Social Work, School of Health Sciences, Faculty of Biology, Medicine and Health; NIHR CLAHRC Greater Manchester<br>Rothwell, Katy; NIHR CLAHRC Greater Manchester, 3rd Floor Mayo Building, Salford Royal NHS Foundation Trust<br>Wilson, Paul; University of Manchester, Alliance Manchester Business School<br>Dumville, Jo C.; University of Manchester, Division of Nursing, Midwifery and Social Work, School of Health Sciences, Faculty of Biology, Medicine and Health; NIHR CLAHRC Greater Manchester<br>Cullum, Nicky; The University of Manchester, Division of Nursing, Midwifery and Social Work, School of Health Sciences, Faculty of Biology, Medicine and Health; NIHR CLAHRC Greater Manchester

**Primary Subject Heading**:	Health services research
Secondary Subject Heading:	Evidence based practice
Keywords:	WOUND MANAGEMENT, Healthcare value, Prevalence survey, Patient centred care, Healthcare quality

SCHOLARONE™ Manuscripts

OPPORTUNITIES FOR BETTER VALUE WOUND CARE: a multi-service, cross sectional survey of complex wounds and their care in a UK community population

Corresponding Author

Trish Gray
Division of Nursing, Midwifery and Social Work; School of Health Sciences;
Faculty of Biology, Medicine and Health.
The University of Manchester, Room 6.332, Jean McFarlane Building,
Oxford Road, Manchester, M13 9PL.
Email: Trish.Gray@manchester.ac.uk
Tel: (+44) (0)161 306 7849

Authors

Trish A Gray,[1,4] Sarah Rhodes,[2,4] Ross A Atkinson,[1,4] Katy Rothwell,[4] Paul Wilson,[3,4] Jo C Dumville,[1,4] Nicky A Cullum.[1,4,5]

Author Affiliations

1 Division of Nursing, Midwifery and Social Work, School of Health Sciences, Faculty of Biology, Medicine and Health, University of Manchester, Manchester, UK

2 Centre for Biostatistics, Faculty of Biology, Medicine and Health, University of Manchester, Manchester, UK

3 Alliance Manchester Business School, University of Manchester, Manchester, UK

4 NIHR CLAHRC Greater Manchester 3rd Floor Mayo Building, Salford Royal NHS Foundation Trust, Stott Lane, Salford, Manchester, UK

5 Research and Innovation Division, Manchester University NHS Foundation Trust, Grafton Street, Manchester, UK

Word Count

Abstract: 285; Manuscript: 3982

For peer review only

Key Words

Health services research, Evidence based practice, Wound management, Healthcare value, Healthcare quality, Prevalence survey, Patient-centred care,

Abstract

Background

Complex wounds impose a substantial health economic burden worldwide. As wound care is managed across multiple settings by a range of healthcare professionals with varying levels of expertise, the actual care delivered can vary considerably and result in the underuse of evidence-based interventions, the overuse of interventions supported by limited evidence and low value healthcare.

Objectives

To quantify the number, type and management of complex wounds being treated over a two-week period of time and to explore variations in care by comparing current practices in wound assessment, prevention and treatment.

Design

A multi-service cross sectional survey.

Setting

This survey spanned eight community services within five Northern England NHS Trusts.

Results

The point prevalence of complex wounds in this community based population was 16.4 per 10,000 (95% confidence interval 15.9 to 17.0). Based on data from 3179 patients, antimicrobial dressings were being used as the primary dressing for 36% of patients with complex wounds. Forty percent of people with leg ulcers either had not received the recommended Doppler-aided ABPI assessment, or it was unclear whether a

For peer review only

For peer review only - http://bmjopen.bmj.com/site/about/guidelines.xhtml

recording had been taken. Thirty-one percent of patients whose most severe wound was a venous leg ulcer were not receiving compression therapy and there was limited use of two-layer compression hosiery. Of patients with a pressure ulcer, 39% were not using a pressure relieving cushion or mattress.

Conclusions

Marked variations were found in care, underuse of evidence-based practices and overuse of practices that are not supported by robust research evidence. Significant opportunities for delivering better value wound care therefore exist. Efforts should now focus on developing strategies to identify, assess and disinvest from products and practices supported by little or no evidence, and enhance the uptake of those that are.

Article Summary

Strengths and limitations of this study

- This cross sectional survey provides robust community-focused population point prevalence estimates for different types of complex wounds.
- This is the first community-focused multi-service survey to capture the wide variation in treatment and care of complex wounds between different NHS trusts.
- The survey is based on wound care provided by community services and may have missed patients only treated by other services providers such as acute or primary care or those self-treating.
- As this was an anonymised survey we were unable to neither conduct any case validation nor validate wound aetiology.

For peer review only

Introduction

Complex wounds, (wounds with superficial, partial or full thickness skin loss healing by secondary intention) such as lower limb diabetic or venous ulcers, pressure ulcers, open trauma and surgical wounds,[1 2] impose a substantial health economic burden worldwide. In the UK, the point prevalence of complex wounds is estimated at 14.7 per 10,000 population, suggesting that approximately 80,000 people in the UK have one or more complex wound at any one time.[3] Whilst the annual cost of managing wounds has been crudely estimated at £3 billion in the UK,[4] $2.85 billion in Australia[5] and $25 billion in the US,[6] the true cost is unknown. The increasing prevalence of complex wounds with age[7] and multimorbidity[8] means that it is difficult to separate the cost of wound care from the cost of caring for people with complex needs.

Wound care is managed across multiple settings by a range of healthcare professionals (HCPs) with varying levels of expertise.[9-13] HCPs are constantly under pressure to make the right choices for their patients, but when faced with an array of wound care products to choose from,[14] inaccessible or limited research evidence to guide decisions[9-14] and silo-based cost-control measures,[15] decisions may not be based on best practice[16 17] and fragmentation of practice and services may occur.[18] With such diversity, it is unlikely that all patients with wounds will have access to good value healthcare.[19] Unwarranted variation in healthcare at a time of rising demand is a concern for health systems globally. It has been estimated that around a third of medical practices are effective or likely to be effective, 50% are of unknown effectiveness and 15% are harmful or unlikely to be beneficial.[20] The cost-effectiveness[21] and value are even less well known.

Value in healthcare is centred on the interests and activities of all stakeholders and is measured by outcomes achieved relative to cost. This perspective shifts the focus on the delivery of care from volume alone to the value gained for patients from the healthcare investments being made e.g. staff time and treatment costs.[19] Every individual should have access to value-based care that considers their needs, preferences and priorities.[19] Gaining maximum healthcare value, given available resources, has been central to discussions and policy changes worldwide in recent years.[22] Decision makers, especially those at policy and organisational levels, are increasingly aiming to maximise patient benefit whilst minimising the opportunity

For peer review only

costs of current healthcare approaches. The term “opportunity cost” refers to the potential for utilised resources to achieve more value elsewhere in the healthcare system.[23] High opportunity costs occur with the overuse of ineffective treatments (or those that make a very small clinical difference), leading to wasted patient benefit and reduced value in the healthcare system. Conversely, underuse of treatments known to be effective also leads to waste.[22]

In some cases, large improvements in the value of healthcare can be made relatively easily by the implementation of evidence based guidelines[14 24 25] and smarter procurement of services and products.[26] We are seeing the development of initiatives focussed on reducing variation and improving value, not only in the UK,[23 26-28] but across the world.[29-31] One campaign launched in 2012, ‘Choosing Wisely’, has been adopted by 12 countries.[32]

The first step to reducing unwarranted variation is to identify: 1) overuse of interventions that do not clearly offer value and 2) underuse of interventions known to offer value. Once such situations have been identified, organisations can start to work towards increased use of effective practices across the relevant populations they are responsible for. To date there have been no such initiatives focused in wound care; a significant yet often neglected area of care and healthcare spend, where there is great potential for better value care. To this end we explored variation in common interventions for complex wounds across eight community services (spanning five NHS Trusts) in the North of England. This project forms part of a wider wound care programme developed by the National Institute for Health Research Collaboration for Leadership in Applied Health Research and Care Greater Manchester (NIHR CLAHRC GM). Given the dearth of basic epidemiological data on chronic or complex wounds in community settings,[33-35] we sought to quantify the number, type and management of complex wounds being treated over a two-week period. We then compared current practices in wound assessment, prevention and treatment with evidence-based recommendations, allowing exploration of variations in care between areas where distinct healthcare organisations provide care to geographically proximal residents. Key treatments of interest included the use of antimicrobial wound dressings, compression for people with venous leg ulcers (VLUs) and the use of

For peer review only - http://bmjopen.bmj.com/site/about/guidelines.xhtml

130 pressure relief for pressure ulcer prevention, as these are specifically highlighted in recent guidelines;[14 24 36 37]

131 [38] as presented in Box 1.

132

133 **Box 1. Key evidence and recommendations related to the assessment, prevention and treatment of complex wounds**

Wound	Key treatments of interest	Guidelines and Recommendations
**Infected (All complex wounds)**	Silver dressings	Insufficient evidence to support the use of silver-containing dressings to promote wound healing or prevent wound infections.[14]
	Honey	Some high quality evidence (based on two randomised controlled trials (RCTs) only) has shown honey to heal partial thickness burns and infected post-operative wounds more quickly than comparators, however, comparators may not be relevant to current practice. Insufficient evidence to support the use of honey in other wounds.[14]
	Iodine	There is insufficient evidence addressing effectiveness and safety for use of iodine to treat or prevent wound infection.[14]
**Venous leg ulcer (VLU)**	Ankle Brachial Pressure Index (ABPI)	Measurement of ABPI should be performed by appropriately trained practitioners to substantiate the presence or absence of peripheral arterial disease (PAD) at initial assessment and to regularly review the use of compression therapy[37]
	Compression therapy	Simple non-adherent dressings and high compression multicomponent bandaging should be used for treating VLU patients with ABPI $\geq$ 0.8. Graduated compression hosiery is recommended to prevent recurrence of VLU.[37]
		Two layer compression stockings are as clinically effective as high compression bandages but more cost effective[35]
	Pentoxifylline	High quality evidence based on systematic review and meta-analysis has found improved VLU healing with the use of pentoxifylline (believed to increase microcirculatory blood flow although exact mechanism of action is unknown) and should be considered in patients with VLU.[37 39]
**Diabetic foot ulcer**	Dressing choice	Insufficient evidence to support the use of any specific dressing. Clinical assessment and patient preference should be taken into consideration whilst the lowest acquisition cost appropriate to the clinical circumstances should be used.[14 36]
	Pressure relief	Offer non-removable casting to offload plantar neuropathic, non-ischaemic, uninfected forefoot and midfoot diabetic ulcers taking into consideration clinical assessment and patient preference. Use pressure redistributing devices and strategies to minimise the risk of pressure ulcers developing.[36]
**Pressure ulcer (PU)**	Dressing choice	Insufficient evidence to support the use of any specific dressing, choice should be determined by the patient's pain, tolerance, location of the ulcer and amount of exudate. A dressing that promotes a warm, moist wound healing environment should be considered for grade 2, 3 and 4 Pus.[14 24]
	Pressure relief	Use high-specification foam mattresses, or consider the use of dynamic support surface if not sufficient. Consider high specification foam or equivalent pressure redistributing cushion for chair or wheelchair use.[24]

134

For peer review only

For peer review only - http://bmjopen.bmj.com/site/about/guidelines.xhtml

Materials and Methods

Project design and participating organisations

A multi-service, cross sectional survey recorded wound prevalence and care for people living with complex wounds across eight community services within five NHS Trusts in the north of England (with a population of 1.9 million). The methods used were based on a previous multi-service complex wound survey undertaken in Leeds, UK.[3]

As most wound care takes place in the community, the project focused on community-based wound care and did not include hospitals or primary care. HCPs working in a range of services (including tissue viability, adult and children's community nursing, podiatry, intermediate care, burns and plastics, specialist leg ulcer and specialist diabetic foot teams,) collected data for consecutive patients over a period of two weeks at each site between June 2015 and September 2016. Face-to-face training and accompanying instructions were provided to improve data accuracy and conformity of responses. Reminders were provided via telephone and email in the week leading to commencement. All services had a helpline number they could call if they had any questions either before or during the process.

Data collection

A structured questionnaire was developed (Appendix 1), largely based on one used successfully in a similar study.[3] The questionnaire was designed to capture the number and nature of wounds and the care provided in terms of workforce, service configuration, assessments used (i.e. ankle brachial pressure index (ABPI)), treatment choices, and product usage. Survey forms were delivered to all clinical areas across the five community NHS Trusts. Data were collected for all patients receiving one or more episodes of wound care for their complex wound(s) from an NHS community service during a two-week period. Forms were completed following the consultation during office time, without the patient present and were anonymised at source. To reduce duplication, forms were completed by the person providing the most hands-on care. This was supported by further local processes, such as placing stickers in patients' clinical notes when a form

For peer review only - http://bmjopen.bmj.com/site/about/guidelines.xhtml
For peer review only

had been completed. Potential duplicates were not removed from the dataset; as data were anonymised we could not be sure whether they were true duplications. This survey was defined as service review by the Trusts' Research and Development Departments and did not require approval from a Research Ethics Committee.

The survey consisted of structured questions relating to patient demographics, wound identification by type, number and severity. The form also asked for more detail about each patient's most severe wound. Thus if a patent had multiple complex wounds, more detailed information (e.g. on wound treatment) was only collected for one wound. For patients with multiple wounds of equal severity, HCPs were asked to focus on the largest wound. Information requested relating to investigations undertaken (e.g. Doppler-aided measurement of ABPI) or treatments provided (e.g. providing pressure relieving equipment) are all specific aspects of care carried out in community settings by the HCPs (and their colleagues) responsible for completing the questionnaire, and it would be expected that such clinical procedures would be recorded in the patient's community health record. The questionnaire had been used previously[3] and was piloted locally prior to commencement of the survey by a range of HCPs. Minor changes were made in the light of feedback received.

Data Analysis

All data were analysed using simple summary statistics; numbers with percentages for categorical data and mean/median values with range for numerical data. Community point prevalence rates per 10,000 population were produced for each wound type, along with 95% confidence intervals (CI). The denominator used for these calculations was 1 935 683 based on total population figures for the five trusts surveyed, taken from Health and Social Care Information Centre 2015 data.

For peer review only

For peer review only - http://bmjopen.bmj.com/site/about/guidelines.xhtml

Results

Description of community complex wound population

Overall we recorded data for 3179 patients with a total of 5632 complex wounds (median number of wounds per patient 1, range 1-24), corresponding to an overall community point prevalence (CPP) of 16.4 per 10,000 (95% CI 15.9 to 17.0). People with complex wounds tended to be elderly (median age 74 years) with at least one comorbidity (median 1.0, range 0-9). Cardiovascular disease was the most frequently reported comorbidity (in 1808 patients; 57%) followed by diabetes (817; 26%) and arthritis (641; 20%). Just under a half of the patients with complex wounds were immobile or walked with difficultly (Table 1) and 494 (31%) of 1613 patients reported as being fully mobile were receiving home visits from a HCP. VLUs were the most prevalent complex wound type (n=612; CPP 3.2 per 10,000; 95% CI 2.9 to 3.4), followed by diabetic foot ulcers (n=488; CPP 2.5 per 10,000; 95% CI 2.3 to 2.7), traumatic wounds (n=428; CPP 2.2 per 10,000; 95% CI 2.0 to 2.4) and pressure ulcers (n=348; CPP 1.8 per 10,000; 95% CI 1.6 to 2.0).

For peer review only

197 **Table 1. Demographic characteristics of patients with at least one complex wound**

Characteristic	
Gender (n=2967)	
Male n (%)	1439 (49)
Female n (%)	1528 (51)
Ethnicity (n=3152)	
White British n (%)	2819 (89)
Other n (%)	336 (11)
Age(n=3120)	
Median (range)	74 (1-107)
**Accommodation (n=3157)**	
Own/rented home n (%)	2728 (86)
Nursing/residential home n (%)	348 (11)
Other n (%)	84 (3)
Number of comorbidities (n=3179)	
Median (range)	1 (0-9)
Continence (n=3029)	
No incontinence n (%)	2487 (82)
Urinary or faecal incontinence or both n (%)	542 (18)
Mobility (n=3141)	
Fully mobile n (%)	1613 (51)
Walks with difficulty n (%)	1091 (35)
Immobile n (%)	437 (14)
**Community point prevalence per 10,000 population for most common wound types n (CPP; 95% CI)**	
Venous leg ulcer	612 (3.2; 2.9 to 3.4)
Diabetic foot ulcer	488 (2.5; 2.3 to 2.7)
Traumatic wound	428 (2.2; 2.0 to 2.4)
Pressure ulcer	348 (1.8; 1.6 to 2.0)

198

199 *Use of wound dressings for all complex wounds*

200 Wound dressings are applied to all types of complex wound. Of the 3038 patients for whom data on the
201 'most severe wound' were provided, 1096 (36%) patients were receiving an antimicrobial primary dressing:
202 383 (13%) a silver dressing and 713 (23%) patients were receiving other antimicrobial dressings such as
203 iodine or honey. There was marked variation in the use of antimicrobial dressings across community services
204 as shown in Figure 1; ranging from 18% of patients in one area to 69% in another. As noted in Box 1, there is
205 currently insufficient evidence to support the use of antimicrobial dressings to promote wound healing[14]. It is
206 highly uncertain that antimicrobial dressings are clinically or cost effective and there is certainly no good
207 evidence that they improve wound healing or other outcomes.[14] For VLUs, the use of anti-microbial
208 dressings is specifically not recommended.[37]

209

For peer review only - http://bmjopen.bmj.com/site/about/guidelines.xhtml

The assessment and treatment of leg ulcers

A leg ulcer was reported as being the most severe wound in 25% (n=770) of patients with a most severe wound selected. For these patients a Doppler-aided ABPI measurement is a crucial part of the assessment process to rule out significant peripheral arterial disease and determine treatment choice and access (Box 1).[37] In total, of those patients with one or more leg ulcer, 19% (n=150) did not have an ABPI recorded in community-held notes. The frequency of ABPI recording varied across services from 10% to 28% and for a further 21% (n=167) this information was either unknown or not reported (ranging from 13% to 22%).

A VLU was reported as being the most severe wound in 570 patients of whom 175 (31%) were recorded as receiving no compression therapy; ranging from 2% to 30% (compression is an effective first-line treatment for venous ulcers[40 41]). Half (n=287; 50%) of those with VLUs were managed with compression bandages, 79 (14%) with compression hosiery and 29 (5%) with a combination of the two (Figure 2). There was limited use of two layer compression hosiery across all areas surveyed despite its known cost-effectiveness relative to compression bandaging.[35] None of the patients with a diagnosis of VLU were prescribed pentoxifylline; a treatment shown to be clinically and cost effective and recommended to promote the healing of VLUs.[37]

Use of support surfaces in those at risk of pressure ulceration

Over one third (35%) of patients with complex wounds were reported as being at risk of pressure ulceration and 348 (11%) had a pressure ulcer at the time of survey. Of the 281 patients whose most severe wound was a pressure ulcer (who are thus at known high risk of further ulceration and should be receiving pressure relief[24]), 109 (39%) patients were reported as not having a pressure relieving cushion or mattress (ranging from 27% in one area to 64% in another; Figure 3). For the 711 patients with a foot ulcer selected as the most severe wound, 40% (n=286) were not receiving any pressure relief for the affected foot (ranging from 31% to 60%).

For peer review only - http://bmjopen.bmj.com/site/about/guidelines.xhtml

Discussion

This study characterised the number and nature of complex wounds being cared for by NHS community services, and the assessments and treatments being used in their management. Our estimate of the point prevalence of complex wounds (16.4 cases per 10,000 population) is consistent with another estimate from the North of England (14.7 per 10,000 population[3]). As these are point prevalence estimates from general populations using census figures as denominators, the estimates are lower than those estimates which focus on specific high-risk populations (such as hospital patients).[1] We found indicators that revealed unwarranted variation in clinical practice across participating services, the underuse of evidence-based interventions (e.g., compression therapy for venous ulcers) and the overuse of interventions supported by limited evidence (e.g., antimicrobial dressings). Overuse of such treatments could incur opportunity cost; drawing resource from the system that cannot be used to confer benefit elsewhere.

Overuse of interventions supported by limited evidence

Across community services, the survey revealed substantial variation in the use of antimicrobial dressings for wound management, ranging from 18% to 69% of all primary dressings used. The unit cost of antimicrobial dressings is often higher than that of their non-antimicrobial counterparts; their use is associated with a high cost for little or no known patient benefit. To illustrate the type of impact of this use we considered silver dressings for which current evidence on the relative effectiveness for preventing or treating wound infection and promoting healing is uncertain.[42 43] Silver dressings cost several times more than their non-silver counterparts. In a conservative scenario where silver dressings are used for a patient over a 2-week period with two dressing changes per week, the dressing cost (using an average cost for a small dressing) would be approximately £32. The cost for a standard foam dressing (of the same size) in the same scenario is approximately five times less (£6.80). Use of silver dressings in this situation (assuming all other aspects of care and outcome are equal) corresponds to an additional £2,520 per 100 patients treated with silver dressings. Tackling overuse is not an easy task and not solely the responsibility of individual HCPs. But despite increased awareness among clinicians, policy makers and the general public during the last 5-10

For peer review only - http://bmjopen.bmj.com/site/about/guidelines.xhtml

years, there have not been widespread changes in policy to address this issue.[44] In wound care specifically, removing silver dressings from a prescribing formulary would offer a partial solution only. There is a clear need for a rational strategy for identifying, assessing and disinvesting from products supported by little or no evidence; ideally this should be delivered at a health system level.[45]

Underuse of evidence-based interventions

Across our population we saw underuse of ABPI in those with leg ulcers (with 19% not having this measure recorded and for 21% it was unclear whether a recording had been taken). Such under-assessment (whether a delay or omission) is likely linked to the underuse of compression therapy also observed. These related issues require urgent attention since compression is a highly efficient and cost-effective treatment for VLU.[37] The first response to this finding is to seek an understanding of the reasoning behind the observed patterns of compression use. For example, underuse may be due to patients waiting for ABPI assessment, or ABPI assessment identifying peripheral arterial disease that may preclude compression use; in other cases, lack of compression may reflect issues with patient adherence to therapy or a shortage of staff with compression bandaging skills. The extent to which current findings can be explained by appropriate contraindications is unclear and requires further investigation. Crucially the reason for such wide variation between relatively local areas also needs further explanation.

Using data from RCTs,[46 47]we conservatively calculate that non-use of compression in those with VLUs will lead to 23 fewer people per 100 patients with VLU healing over 12 months compared with use of compression. Using an estimated mean cost per VLU episode of £1800[35] we can estimate an annual increased cost of £41,400 per 100 VLU patients not using compression. However, we could tentatively also consider non-use of compression as a proxy for sub-optimal care. If we apply the recent Rightcare[28] estimated cost savings associated with optimised VLU care (including high compression use), savings could be as high as £118,979 per 100 VLU patients. While here we have focused on cost impacts, it is important to consider the improved health-related quality of life associated with a healed wound as well as the reduced

For peer review only - http://bmjopen.bmj.com/site/about/guidelines.xhtml
For peer review only

financial burden on patients, especially those who are unable to work or require time away from work to attend clinic appointments.

There is also an apparent underuse of pentoxifylline, a xanthine derivative used to treat muscle pain in people with peripheral arterial disease, but also believed to increase microcirculatory blood flow in patients with VLU. Uptake is low despite evidence from 12 RCTs included in a systematic review[39] that it increases ulcer healing both with and without compression. The reasons for this underuse are being explored further but are likely to include: lack of awareness of the drug and its effects amongst both community GPs and nurses; relative lack of nurse prescribers able to prescribe pentoxifylline and reluctance to prescribe for an off-licence indication (particularly in people who may be taking several other medicines for comorbidities). It is also worth noting that the drug is low cost but not marketed because the patent is long expired. It is unclear whether this treatment is used more widely outside the UK. Using published relative effectiveness estimates and related costs,[48] we have estimated that use of pentoxifylline alongside compression could result in cost savings of over £40,000 per 100 VLU patients treated. Combined increases in the use of ABPI, cost-effective compression therapy and greater pentoxifylline use in the treatment of those with venous leg ulceration is likely to see a cumulative increase in improved patient outcomes, experiences and reduced health care costs.

Similar exploration is required in relation to the potential underuse of pressure relieving equipment suggested by our study, in order to understand the true scope for improvement. In all cases, further work is required to identify the factors that underpin clinical decision making and behaviour in these areas. An understanding of such factors would support the identification and selection of appropriate behaviour change techniques and implementation strategies, targeted at modifying these behaviours and generating improvement and thus value in healthcare. Having a pressure ulcer is prognostic for further pressure ulceration, thus those with an ulcer are considered at risk and should receive pressure relieving interventions such as support surfaces. Even accounting for the initial cost of purchasing a device given the large cost associated with healing a pressure ulcer (estimated cost for healing a Grade 2 pressure ulcer = £5,241),[49] we

For peer review only - http://bmjopen.bmj.com/site/about/guidelines.xhtml
For peer review only

conservatively calculate that the use of support surfaces to prevent pressure ulceration could save at least £38,000 per 100 at-risk patients. Again those with a pressure ulcer are also known to have worse health-related quality of life than others with similar co-morbidities.[50] These are not isolated examples of underuse: a US study found that patients with acute or chronic conditions (that represented the leading causes of illness, death and utilization of healthcare) received only 55% of recommended care[51] and similarly the CareTrack study found that Australians received appropriate care in only 57% of 35,573 eligible healthcare encounters.[52] However, this is the first work that has revealed so clearly, variation in the use of interventions for VLUs where there are corresponding guideline-based recommendations. Failure to deliver best practice is often a result of poor execution or lack of widespread adoption of best care processes.[53] International interest in research translation and quality improvement reflects the growing recognition of the slow and inconsistent uptake of effective healthcare practices worldwide.[54]

Disinvestment and implementation of improvement initiatives to promote better value care

Local variation in product choice between organisations is shown clearly in the work presented. This highlights the huge scope for better value care in both the assessment and treatment of complex wounds; value could be released by disinvestment in some areas with savings being focused on areas of underuse identified here. Further work should be undertaken to understand the factors that underpin decision making around treatment use, with a particular focus on exploring the motivations to use expensive treatments with limited evidence at a time when the NHS faces significant resource constraints. Working with our service partners, we plan to address the observed local practice variations through a programme of improvement work undertaken as part of NIHR CLAHRC GM. However, we recognise that this process needs to be replicated across the health system as a whole if overuse and underuse are to be addressed fully. As we have stated above, there is a clear need for a national strategy for identifying, assessing and disinvesting from products and practices supported by little or no evidence.[45] A model for taking a systematic approach to disinvestment already exists in the form of the SHARE programme in Australia. This model enables clinicians, managers and policy makers to manage the process from identifying the need for disinvestment to

For peer review only - http://bmjopen.bmj.com/site/about/guidelines.xhtml
For peer review only

implementing the change and evaluating outcomes.[55] Similar efforts are required in the UK if practice variations are to be addressed.

Strengths and limitations

This cross sectional survey provides robust community-focused population point prevalence estimates for different types of complex wounds and is the first multi-service survey to capture the wide variation in treatment and care of complex wounds between different NHS Trusts. There are a few limitations to our study. Firstly, the study looked specifically at patients receiving care from NHS community services and did not include people whose wound care was delivered by hospitals, primary care, or other care providers, and also those who may have been self-caring at this time. While this prevents us from comparing the characteristics of people with and without complex wounds at the time of the survey, collection of these data would have been too resource intensive for participating services and we know from previous work that the vast majority of people with wounds are cared for in community settings.[1] Secondly, we only scrutinised the patient information readily available to community staff and did not examine wider (e.g., GP) patient records. However given that we were looking for information regarded as crucial to the management of people with complex wounds, any omission of information from community records risks suboptimal clinical decision making. Thirdly, it is also conceivable that services failed to complete a survey form for each patient they saw with a complex wound during the survey period. However, both the level of engagement from services and the similarity between the estimated CPP obtained here and that found in a previous but smaller survey in Leeds,[3] suggest the impact of this is likely to be negligible. Finally, as this was an anonymised survey we were unable to neither conduct any case validation nor validate wound aetiology. Consequently, data presented reflects the treating HCP’s assessment.

Conclusions

This survey adds important robust epidemiological data to the complex wound literature where existing prevalence data have been found to be limited when systematically reviewed.[1] We also highlight the over-

For peer review only - http://bmjopen.bmj.com/site/about/guidelines.xhtml

and underuse of services and treatments and reflect the issues emphasised in a number of reports relating to Better Value Healthcare.[19 26 29] These findings suggest significant opportunities for delivering better value wound care exist. Efforts should now focus on developing strategies to identify, assess and disinvest from products and practices supported by little or no evidence and enhance the uptake of those that are.

Figure Legends

Figure 1. Proportion of complex wounds for which primary dressing contained antimicrobials: the other anti-microbial dressing group maps to the same section of the British National Formulary and includes dressings such as Polyhexanide polyhexamethylene biguanide (bars represent included community services), *Number of patients per community service ranged from 172 to 655*

Figure 2. Highest level of compression used for patients with venous leg ulcers selected as most severe wound (bars represent included community services). *Number of patients per community service ranged from 14 to 151*

Figure 3. Proportion of patients using pressure relieving mattresses or cushions (bars represent included community services). *Number of patients per community service ranged from 10 to 63*

References

1. Cullum N, Buckley H, Dumville J, et al. Wounds Research for Patient Benefit: a 5 year programme of research. *Programme Grants Appl Res* 2016;4(13)
2. Vowden K. Complex wound or complex patient. *internursecom* 2005;10(Sup2):S6-14.
3. Hall J, Buckley HL, Lamb KA, et al. Point prevalence of complex wounds in a defined United Kingdom population. *Wound Repair Regen* 2014;22(6):694-700. doi: 10.1111/wrr.12230 [published Online First: 2014/09/17]
4. Guest JF, Ayoub N, McIlwraith T, et al. Health economic burden that wounds impose on the National Health Service in the UK. *BMJ Open* 2015;5(12):e009283. doi: 10.1136/bmjopen-2015-009283
5. Graves N, Zheng H. Modelling the direct health care costs of chronic wounds in Australia. *Wound Practice and Research* 2014; 22(1).
6. Sen CK, Gordillo GM, Roy S, et al. Human skin wounds: a major and snowballing threat to public health and the economy. *Wound Repair Regen* 2009;17(6):763-71. doi: 10.1111/j.1524-475X.2009.00543.x
7. WHO. Global Health and Aging: National Institute on Aging, National Institutes of Heath, US Department of Health and Human Services, World Health Organisation; 2011 [Available from: http://www.who.int/ageing/publications/global_health.pdf.
8. Gould L, Abadir P, Brem H, et al. Chronic wound repair and healing in older adults: current status and future research. *J Am Geriatr Soc* 2015;63(3):427-38. doi: 10.1111/jgs.13332
9. Corbett LQ. Wound Care Nursing: Professional Issues and Opportunities. *Advances in Wound Care* 2011;1(5):189-93.
10. Harrison MB, Graham ID, Lorimer K, et al. Nurse clinic versus home delivery of evidence-based community leg ulcer care: a randomized health services trial. *BMC Health Serv Res* 2008;8:243. doi: 10.1186/1472-6963-8-243
11. Medical Advisory Secretariat. Community-based care for chronic wound management: an evidence-based analysis. *Ontario Health Technology Assessment Series* 2009;9(18)
12. Moore Z, Butcher G, Corbett LQ, et al. AAWC, AWMA, EWMA Position Paper: Managing Wounds as a Team *J Wound Care* 2014;23:S1-S38.
13. Whitlock E, Morcom J, Spurling G, et al. Wound care costs in general practice - a cross-sectional study. *Aust Fam Physician* 2014;43(3):143-6.
14. NICE. Chronic wounds: advanced wound dressings and antimicrobial dressings: Evidence summary [ESMPB2]: National Institute for Health and Care Excellence, 2016.

For peer review only - http://bmjopen.bmj.com/site/about/guidelines.xhtml

15. Erstad BL. Value-Based Medicine: Dollars and Sense. *Crit Care Med* 2016;44(2):375-80. doi: 10.1097/CCM.0000000000001559
16. Gabbay J, le May A. Evidence based guidelines or collectively constructed "mindlines?" Ethnographic study of knowledge management in primary care. *BMJ* 2004;329(7473):1013. doi: 10.1136/bmj.329.7473.1013
17. Thompson C, Cullum N, McCaughan D, et al. Nurses, information use, and clinical decision making--the real world potential for evidence-based decisions in nursing. *Evid Based Nurs* 2004;7(3):68-72.
18. Enthoven AC. Integrated delivery systems: the cure for fragmentation. *Am J Manag Care* 2009;15(10 Suppl):S284-90.
19. Porter ME. What is value in health care? *N Engl J Med* 2010;363(26):2477-81. doi: 10.1056/NEJMp1011024
20. Prasad V, Vandross A, Toomey C, et al. A decade of reversal: an analysis of 146 contradicted medical practices. *Mayo Clin Proc* 2013;88(8):790-8. doi: 10.1016/j.mayocp.2013.05.012
21. Productivity Commission. Impacts of Advances in Medical Technology in Australia. Research Report 2005 [Available from: http://www.pc.gov.au/inquiries/completed/medical-technology. (accessed May 2017) accessed May 2017 2017.
22. WHO. Appropriateness in Health Care Services. In: WHO Regional Office for Europe, ed. Copenhagen, Denmark World Health Organisation, 2000.
23. Muir Gray JA. How to Get Better Value Healthcare Second ed. Oxford, UK: Offox Press for Better Value Healthcare Ltd 2011.
24. NICE. Pressure ulcers: prevention and management Clinical guideline [CG179]: National Institute for Health and Care Excellence, 2014.
25. Ubbink DT, Santema TB, Stoekenbroek RM. Systemic wound care: a meta-review of cochrane systematic reviews. *Surg Technol Int* 2014;24:99-111.
26. Alderwick H, Robertson R, Appleby J, et al. Better value in the NHS: The role of changes in clinical practice. London, UK: The King's Fund, 2015.
27. Cripps M. NHS Right Care – expanding the approach in the context of delivering the Five Year Forward View: NHS England; 2015 [Available from: https://www.england.nhs.uk/wp-content/uploads/2015/12/nqb-oct15-5.pdf (accessed April 2017) accessed 24th April 2017.
28. NHS RightCare. NHS RightCare scenario: The variation between sub-optimal and optimal pathways. Betty's story: Leg ulcer wound care NHS England; 2017 [Available from: https://www.england.nhs.uk/rightcare/wp-content/uploads/sites/40/2017/01/nhs-rightcare-bettys-story-narrative-full.pdf (accessed May 2017).
29. Levinson W, Kallewaard M, Bhatia RS, et al. 'Choosing Wisely': a growing international campaign. *BMJ Qual Saf* 2015;24(2):167-74. doi: 10.1136/bmjqs-2014-003821
30. ABIM Foundation. Choosing Wisely Philadelphia: ; 2017 [Available from: http://www.choosingwisely.org/ (accessed June 2017) accessed 18th May 2017 2017.
31. Canadian Medical Association. Choosing Wisley Canada University of Toronto 2017 [Available from: http://www.choosingwiselycanada.org/ (accessed May 2017).
32. Bhatia RS, Levinson W, Shortt S, et al. Measuring the effect of Choosing Wisely: an integrated framework to assess campaign impact on low-value care. *BMJ Qual Saf* 2015;24(8):523-31. doi: 10.1136/bmjqs-2015-004070
33. Hodgson R, Allen R, Broderick E, et al. Funding source and the quality of reports of chronic wounds trials: 2004 to 2011. *Trials* 2014;15:19. doi: 10.1186/1745-6215-15-19
34. Lazarus GS, Kirsner RS, Zenilman J, et al. Clinical interventions for venous leg ulcers: Proposals to improve the quality of clinical leg ulcer research. *Wound Repair Regen* 2016;24(5):767-74. doi: 10.1111/wrr.12466
35. Ashby RL, Gabe R, Ali S, et al. Clinical and cost-effectiveness of compression hosiery versus compression bandages in treatment of venous leg ulcers (Venous leg Ulcer Study IV, VenUS IV): a randomised controlled trial. *Lancet* 2014;383(9920):871-9. doi: 10.1016/S0140-6736(13)62368-5
36. NICE. Diabetic foot problems: prevention and management [NG19]: National Institute for Health and Care Excellence, 2015.
37. SIGN. Management of chronic venous leg ulcers: A national clinical quideline (120): Scottish Intercollegiate Guidelines Network, 2010.
38. Kelly J, Clifton E, Fearns N, Harbour J, Heller-Murphy S, Herbert P, Macpherson K, Myles S, Santo V, Wilson L Antimicrobial wound dressings (AWDs) for chronic wounds. Health Technology Assessment report 13: Health Improvement Scotland, 2015.
39. Jull AB, Arroll B, Parag V, et al. Pentoxifylline for treating venous leg ulcers. *Cochrane Database Syst Rev* 2012;12:CD001733. doi: 10.1002/14651858.CD001733.pub3
40. Nelson EA, Bell-Syer SE. Compression for preventing recurrence of venous ulcers. *Cochrane Database Syst Rev* 2014(9):CD002303. doi: 10.1002/14651858.CD002303.pub3
41. O'Meara S, Cullum N, Nelson EA, et al. Compression for venous leg ulcers. *Cochrane Database Syst Rev* 2012;11:CD000265. doi: 10.1002/14651858.CD000265.pub3

For peer review only - http://bmjopen.bmj.com/site/about/guidelines.xhtml

42. Storm-Versloot MN, Vos CG, Ubbink DT, et al. Topical silver for preventing wound infection. *Cochrane Database Syst Rev* 2010(3):CD006478. doi: 10.1002/14651858.CD006478.pub2
43. Vermeulen H, van Hattem JM, Storm-Versloot MN, et al. Topical silver for treating infected wounds. *Cochrane Database Syst Rev* 2007(1):CD005486. doi: 10.1002/14651858.CD005486.pub2
44. Brownlee S, Chalkidou K, Doust J, et al. Evidence for overuse of medical services around the world. *Lancet* 2017 doi: 10.1016/S0140-6736(16)32585-5
45. Prasad V, Ioannidis JP. Evidence-based de-implementation for contradicted, unproven, and aspiring healthcare practices. *Implement Sci* 2014;9:1. doi: 10.1186/1748-5908-9-1
46. Ashby RL, Gabe R, Ali S, et al. VenUS IV (Venous leg Ulcer Study IV) - compression hosiery compared with compression bandaging in the treatment of venous leg ulcers: a randomised controlled trial, mixed-treatment comparison and decision-analytic model. *Health Technol Assess* 2014;18(57):1-293, v-vi. doi: 10.3310/hta18570
47. Morrell CJ, King B, Brereton L. Community-based leg ulcer clinics: organisation and cost-effectiveness. *Nurs Times* 1998;94(9):51-4.
48. Iglesias CP, Birks Y, Nelson EA, et al. Quality of life of people with venous leg ulcers: a comparison of the discriminative and responsive characteristics of two generic and a disease specific instruments. *Qual Life Res* 2005;14(7):1705-18.
49. Bennett G, Dealey C, Posnett J. The cost of pressure ulcers in the UK. *Age Ageing* 2004;33(3):230-5. doi: 10.1093/ageing/afh086
50. Essex HN, Clark M, Sims J, et al. Health-related quality of life in hospital inpatients with pressure ulceration: assessment using generic health-related quality of life measures. *Wound Repair Regen* 2009;17(6):797-805. doi: 10.1111/j.1524-475X.2009.00544.x
51. McGlynn EA, Asch SM, Adams J, et al. The quality of health care delivered to adults in the United States. *N Engl J Med* 2003;348(26):2635-45. doi: 10.1056/NEJMsa022615
52. Runciman WB, Hunt TD, Hannaford NA, et al. CareTrack: assessing the appropriateness of health care delivery in Australia. *Med J Aust* 2012;197(2):100-5.
53. Berwick DM, Hackbarth AD. Eliminating waste in US health care. *JAMA* 2012;307(14):1513-6. doi: 10.1001/jama.2012.362
54. Glasziou P, Straus S, Brownlee S, et al. Evidence for underuse of effective medical services around the world. *Lancet* 2017 doi: 10.1016/S0140-6736(16)30946-1
55. Harris C, Green S, Ramsey W, et al. Sustainability in Health care by allocating resources effectively (SHARE) 1: introducing a series of papers reporting an investigation of disinvestment in a local healthcare setting. *BMC Health Serv Res* 2017;17(1):323. doi: 10.1186/s12913-017-2210-7

Funding

This project was funded by the National Institute for Health Research Collaboration for Leadership in Applied Health Research and Care (NIHR CLAHRC) Greater Manchester. The NIHR CLAHRC Greater Manchester is a partnership between providers and commissioners from the NHS, industry and the third sector, as well as clinical and research staff from the University of Manchester. The views expressed in this article are those of the author(s) and not necessarily those of the NHS, NIHR or the Department of Health.

For peer review only

For peer review only - http://bmjopen.bmj.com/site/about/guidelines.xhtml

Contributors

NC and JD conceived the idea and design for the overall project, TG, KR and PW contributed to further development of the study design, TG and KR conducted stakeholder consultation with practitioners, SR was responsible for data analysis, RA and JD contributed to data analysis. TG created the original draft of the manuscript. All authors contributed to the interpretation of study findings, critical revision of the manuscript for important intellectual content and approval of the final manuscript.

Acknowledgements

The authors are grateful to the healthcare professionals, from NHS partner organisations, involved in data collection, for giving their time and enthusiastically supporting this project. The authors would also like to thank Wanda Russell (NIHR CLAHRC GM Research Fellow at the time of this project) for her role in conducting stakeholder consultation with practitioners and data collection and Louise Hussey, Research Fellow, for her contribution to data analysis.

Competing interests

None declared.

Data sharing statement

Requests for access to data should be addressed to the corresponding author.

For peer review only

For peer review only - http://bmjopen.bmj.com/site/about/guidelines.xhtml

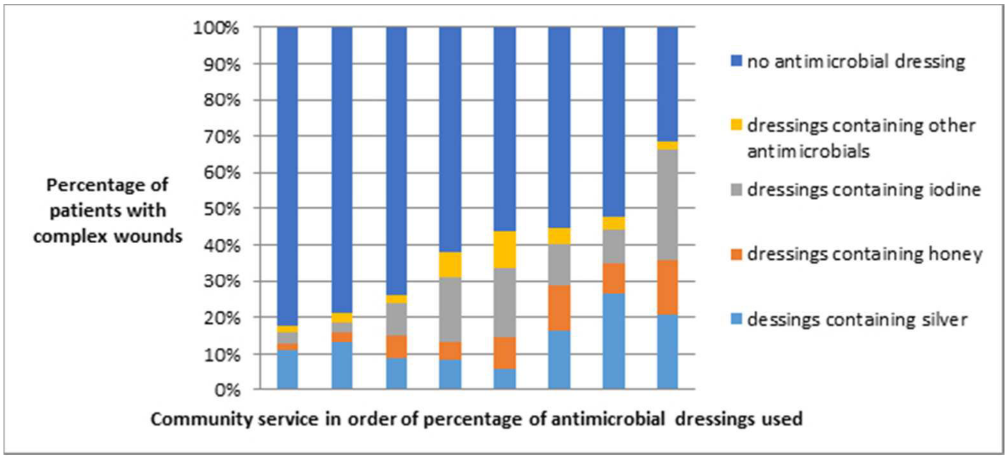

52x23mm (300 x 300 DPI)

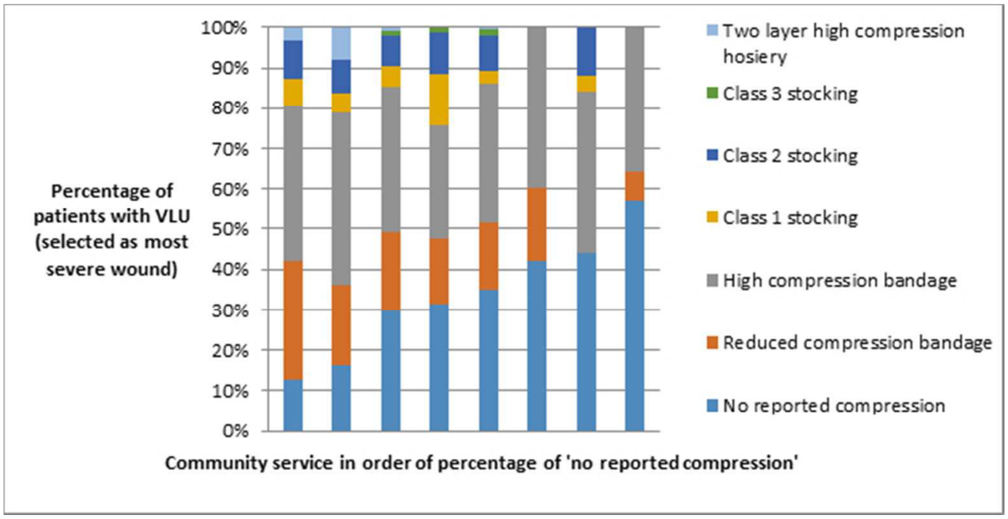

52x27mm (300 x 300 DPI)

For peer review only

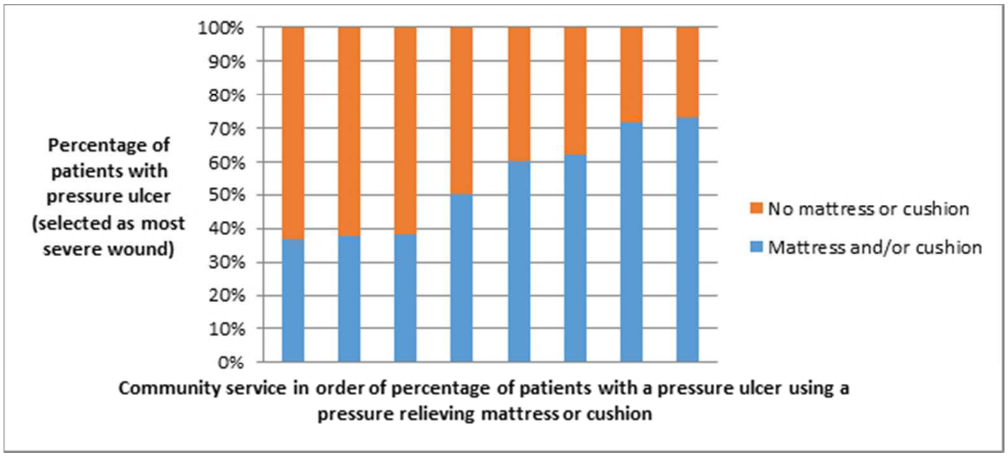

52x23mm (300 x 300 DPI)

For peer review only

Office use only: ID number ☐☐☐☐

The University of Manchester

National Institute for Health Research
Collaboration for Leadership in Applied Health Research and Care (CLAHRC) Greater Manchester

A Survey of Complex Wounds and their Care in the Community

Patient Data Capture Form

Date this form was completed: ☐☐ / ☐☐ / ☐☐☐☐
day *month* *year*

This form is to be completed by the NHS health care professional (from the participating NHS community-based services) delivering most of the hands on wound care for a person with one or more complex wounds. <u>Please aim to ensure that only one form is completed per patient</u>

For peer review only

Section 1:

1.1 What is your main clinical role currently? *(Please cross (x) one option)*

District Nurse/community nurse ☐ Nurse Specialist ☐

Podiatrist ☐ Other ☐

If 'Other' please state: ☐

1.2 What is your NHS Agenda for Change band (as the health care professional from the participating NHS community-based services delivering most of the hands on wound care for this patient)? *(Please cross (x) one option)*

Band 3 ☐ Band 4 ☐ Band 5 ☐ Band 6 ☐ Band 7 ☐ Band 8a ☐

Other ☐ If 'Other' please state: ☐

1.3 Currently, in which location does this patient receive <u>most</u> of their hands on wound care? *(Please cross (x) one option)*

Patient's home ☐ Community clinic/heath centre/GP practice ☐ Nursing home ☐

Residential home ☐ Hospice ☐ Prison ☐

Other ☐ If 'Other' please state: ☐

1.4 How many wound related consultations (with all professionals involved) has THIS patient had in the last seven days?: ☐☐

1.5 Duration of the most recent consultation: ☐☐☐ minutes

1.6 Are any other health professionals involved in deciding or planning wound care for this person? ***(Please cross (x) one option)***

Yes ☐ **No** ☐ Don't know ☐

↓

1.7 If Yes who are they? ***(please cross (x) all that apply)*****:**

GP ☐ Tissue viability nurse ☐ Other wound care specialist ☐

Podiatrist ☐ District nurse ☐

Other ☐ If 'Other' please state: ☐

Section 2: Patient details

2.1 Patient's DOB: dd ☐☐ mm ☐☐ yyyy ☐☐☐☐

2.2 Patient's gender: male ☐ female ☐

2.3 Ethnicity:

White British ☐	White Irish ☐	White Other ☐	Black African ☐	Black Caribbean ☐	Black Other ☐
Asian Indian ☐	Asian Pakistani ☐	Asian Bangladeshi ☐	Asian Other ☐	White and Black Caribbean ☐	White and Black African ☐
White and Asian ☐	Other mixed background ☐	Chinese ☐	Other ☐		

2.4 Please state location of patient's GP ***(Please cross (x) one option)***

Central Manchester ☐ South Manchester ☐ Salford ☐ Oldham ☐

Bury ☐ Heywood, Middleton, Rochdale ☐ Trafford ☐

Other location ☐ If other, please state: ☐

Don't know ☐

For peer review only - http://bmjopen.bmj.com/site/about/guidelines.xhtml

2.5 What type of accommodation best describes where this patient currently lives? *(Please cross (x) one option)*

Own / rented home ☐ Nursing home ☐ Residential home ☐

No fixed abode ☐ Prison ☐

Other ☐ If 'Other' please state: ☐

2.6 What co-morbidities does this patient have? *(please cross (x) all that apply)*:

**Cardiovascular disease (CVD)**	e.g. Hypertension (high blood pressure, high BP), Myocardial infarction (MI, heart attack, IHD), Angina (IHD), Heart failure (CCF, CHF, HF, LVF)	☐
**Peripheral Arterial Disease**	e.g. Ischaemia (leg/foot), Claudication (intermittent claudication), Rest pain, Arteriopathy/angiopathy, Aneurysm including aortic aneurysm	☐
**Diabetes (DM)**	e.g. Insulin dependent DM (IDDM, Type I diabetes), Non insulin dependent DM (NIDDM, Type II diabetes)	☐
**Arthritis**	e.g. Rheumatoid arthritis (RA), Osteoarthritis (OA), Non rheumatoid arthritis	☐
**Orthopaedics**	e.g. Fractured neck of femur (# NOF, # hip, fractured hip, broken hip), Other fracture/break, Elective orthopaedic	☐
**Airways**	e.g. Chronic obstructive airways/pulmonary disease (COAD, COPD), Chronic bronchitis, Emphysema, Asthma	☐
**Neurological**	e.g. Parkinson's disease, Multiple sclerosis (MS), Epilepsy (fits, Grand mal, Petit mal, tonic-clonic, seizure), Spinal Injury	☐
**Stroke**	e.g. (cerebrovascular accident, CVA, transient ischaemic attack, TIA)	☐
**Cancer**	e.g. any cancer diagnosis	☐

2.7 Patient's Continence *(please cross (x) all that apply)*:

No incontinence ☐ Urinary incontinence ☐ Faecal Incontinence ☐

2.8 Patient mobility *(please cross (x) one option)*:

Patient walks freely ☐ Patient walks with difficultly ☐ Patient is immobile ☐

For peer review only

2.9 In your judgement is this patient at risk of pressure ulceration? *(please cross (x) one option):*

Yes ☐ No ☐ Don't know ☐

2.10 If yes is their pressure ulcer risk documented in their notes/care plan? *(please cross (x) one option)*:

Yes ☐ No ☐ Don't know ☐

Section 3: Current complex wounds

3.1 Please record the number of wounds (by wound type) that this patient has.

**Wound type:**		**Number of wound of this type (please circle one number for each type or leave blank if 0):**							
**Foot ulcer in patient with diabetes**	**RIGHT foot**	1	2	3	4	5	6	7	8 or more
	**LEFT foot**	1	2	3	4	5	6	7	8 or more
**Foot ulcer in patient without diabetes**	**RIGHT foot**	1	2	3	4	5	6	7	8 or more
	**LEFT foot**	1	2	3	4	5	6	7	8 or more
**Venous leg ulcer**	**RIGHT leg**	1	2	3	4	5	6	7	8 or more
	**LEFT leg**	1	2	3	4	5	6	7	8 or more
**Arterial leg ulcer**	**RIGHT leg**	1	2	3	4	5	6	7	8 or more
	**LEFT leg**	1	2	3	4	5	6	7	8 or more
**Arterial/venous (mixed aetiology) leg ulcer**	**RIGHT leg**	1	2	3	4	5	6	7	8 or more
	**LEFT leg**	1	2	3	4	5	6	7	8 or more
**Pressure ulcer**		1	2	3	4	5	6	7	8 or more

For peer review only

**Dehisced surgical wound currently healing by secondary intention**	1	2	3	4	5	6	7	8 or more
**Wound resulting from excision of pilonidal sinus**	1	2	3	4	5	6	7	8 or more
**Wound resulting from excision of peri-anal abscess**	1	2	3	4	5	6	7	8 or more
**Other surgical wound healing by secondary intention**	1	2	3	4	5	6	7	8 or more
**Traumatic wound (e.g. result of road traffic accident)**	1	2	3	4	5	6	7	8 or more
**Fungating carcinoma**	1	2	3	4	5	6	7	8 or more
**Burn**	1	2	3	4	5	6	7	8 or more

**Other wound type - please describe:**	**Number of wound of this type (please circle):**							
	1	2	3	4	5	6	7	8 or more
	1	2	3	4	5	6	7	8 or more
	1	2	3	4	5	6	7	8 or more

For peer review only

Section 4: Treatment of complex wounds

We now want to collect information on the treatment of this patient's complex wound(s). If the patient has more than one complex wound (of any type) please now focus on the one wound you consider to be the worst/most severe (if there are more than one wound of equal severity choose any one of these wounds and complete the following information for just this wound).

We will refer to the wound for which the following data are collected as the selected wound.

4.1 Please record what type of wound the selected wound is (as noted above - this is the most severe/wound and therefore the one you are focusing on in this section). ***Please cross (x) one option.***

Foot ulcer in patient with diabetes ☐ Foot ulcer in patient without diabetes ☐

Venous leg ulcer ☐ Arterial leg ulcer ☐

Arterial/venous leg ulcer ☐ Pressure ulcer ☐

Dehisced surgical wound currently healing by secondary intention ☐ Wound resulting from excision of pilonidal sinus ☐

Wound resulting from excision of peri-anal abscess ☐ Other surgical wound healing by secondary intention ☐

Traumatic wound (e.g. result of road traffic accident) ☐ Fungating carcinoma ☐

Burn ☐

Other wound type ☐ If 'Other' please state: ____________

4.2 Please note the location/area most affected by the selected wound. ***Please cross (x) one option.***

head/neck ☐ arm ☐ hand ☐ breast ☐

abdomen ☐ back ☐ sacrum ☐ natal cleft ☐

buttock ☐ peri-anal ☐ perineum ☐ leg ☐

foot ☐ Other ☐ If 'Other' please state: ____________

Please record the following details for the selected wound. ***Please cross (x) one option.***

4.3 Exudate level:

High ☐ Moderate/ medium ☐ Low ☐ None ☐

4.4 Malodour:

Yes ☐ No ☐

For peer review only - http://bmjopen.bmj.com/site/about/guidelines.xhtml

4.5 For the selected wound, which wound dressings are being used currently? *(Refer to the list of dressings in the guidelines and insert the corresponding code in each of the boxes)*:

Primary dressing ☐☐ Secondary dressing ☐☐

Please record any other wound treatments for the selected wound:

4.6 Drugs/medicines: *(please cross (x) all that apply)*

Antibiotics for wound infections ☐ Pentoxifylline ☐

Topical steroids ☐ Analgesics ☐

Other ☐ If 'Other' please state: ☐

4.7 For the selected wound, the bandage currently used: (*Please cross (x) one option).*

N/A ☐ 4 layer compression bandage ☐

Short stretch bandage (e.g. Actico/Comprilan) ☐ 3 layer reduced compression bandage ☐

2 layer compression bandage (e.g. K2) ☐ Non-compression bandage (plus wadding) ☐

Dressing retention bandage (e.g. K-band) ☐

Other ☐ If 'Other' please state: ☐

4.8 Hosiery (stockings) for the leg affected by the selected wound: (*Please cross (x) one option).*

N/A ☐ Standard class 1 ☐

Standard class 2 ☐ Standard class 3 ☐

Made to measure Class 1 ☐ Made to measure Class 2 ☐

Made to measure Class 3 ☐ Standard two layer compression hosiery (aiming to deliver 40mmhg) ☐

Made to measure two layer compression hosiery (aiming to deliver 40mmhg) ☐

Other ☐ If 'Other' please state: ☐

For peer review only

Please record any equipment used relating to the selected wound:

4.9 Pressure relief and mobility: ***(please cross (x) all that apply)***

Pressure relieving	**mattresses:**	**cushions:**
High specification foam replacement e.g. MSS gilde	☐	☐
Air alternating replacement e.g. Quattro deep cell prime	☐	☐
Air alternating overlay e.g. Alpha excel	☐	☐
Repose (overlay system)	☐	☐
Foam overlay	☐	☐

Crutches ☐

Wheelchair ☐

Other ☐

If 'Other' please state:

4.10 Footwear/Orthotics/Foot pressure reliving equipment: ***(please cross (x) all that apply)***

Prescribed footwear ☐

Footwear adaptation ☐

Insoles ☐

Heel pressure relief ☐
e.g. Repose Heel Trough, PROFO boot

Other ☐ If 'Other' please state:

4.11 Other therapies for the selected wound: ***(please cross (x) all that apply)***

Hyperbaric oxygen therapy ☐

Physiotherapy ☐

Occupational therapy ☐

Negative pressure wound therapy ☐

Other ☐ If 'Other' please state:

For peer review only

4.12 Treatment objectives for the selected wound: ***(please cross (x) all that apply)***

Debridement ☐
Absorption (removal of exudate) ☐
Hydration (keep the wound moist) ☐
Microbial load management ☐
Odour Management ☐
Reduce overgranulation ☐
Encourage granulation ☐
Protection ☐
Other ☐
If 'other' please state: ☐

4.13 If the selected wound is a PRESSURE ULCER record its category here (otherwise leave blank). (*Please cross (x) one option).*

I ☐ II ☐ III ☐ IV ☐ Unstageable ☐ Not applicable ☐

If the selected wound is a LEG ULCER please record the following (otherwise leave blank):

4.14 Is a Doppler ABPI for the leg with the selected wound recorded in the notes? (*Please cross (x) one option).*

Yes ☐ No ☐ Don't know ☐

4.15 If yes – please note the ABPI measure here:

☐☐ • ☐☐

For peer review only

BMJ Open

OPPORTUNITIES FOR BETTER VALUE WOUND CARE: a multi-service, cross sectional survey of complex wounds and their care in a UK community population

Journal:	*BMJ Open*
Manuscript ID	bmjopen-2017-019440.R2
Article Type:	Research
Date Submitted by the Author:	09-Feb-2018
Complete List of Authors:	Gray, Trish; University of Manchester, Division of Nursing, Midwifery and Social Work, School of Health Sciences, Faculty of Biology, Medicine and Health; NIHR CLAHRC Greater Manchester<br>Rhodes, Sarah; University of Manchester, Centre for Biostatistics, Faculty of Biology, Medicine and Health; NIHR CLAHRC Greater Manchester<br>Atkinson, Ross; University of Manchester, Division of Nursing, Midwifery and Social Work, School of Health Sciences, Faculty of Biology, Medicine and Health; NIHR CLAHRC Greater Manchester<br>Rothwell, Katy; NIHR CLAHRC Greater Manchester, Salford Royal NHS Foundation Trust<br>Wilson, Paul; University of Manchester, Alliance Manchester Business School<br>Dumville, Jo C.; University of Manchester, Division of Nursing, Midwifery and Social Work, School of Health Sciences, Faculty of Biology, Medicine and Health; NIHR CLAHRC Greater Manchester<br>Cullum, Nicky; The University of Manchester, Division of Nursing, Midwifery and Social Work, School of Health Sciences, Faculty of Biology, Medicine and Health; NIHR CLAHRC Greater Manchester
**Primary Subject Heading**:	Health services research
Secondary Subject Heading:	Evidence based practice
Keywords:	WOUND MANAGEMENT, Healthcare value, Prevalence survey, Patient centred care, Healthcare quality

SCHOLARONE™
Manuscripts

OPPORTUNITIES FOR BETTER VALUE WOUND CARE: a multi-service, cross sectional survey of complex wounds and their care in a UK community population

Corresponding Author

Trish Gray
Division of Nursing, Midwifery and Social Work; School of Health Sciences;
Faculty of Biology, Medicine and Health.
The University of Manchester, Room 6.332, Jean McFarlane Building,
Oxford Road, Manchester, M13 9PL.
Email: Trish.Gray@manchester.ac.uk
Tel: (+44) (0)161 306 7849

Authors

Trish A Gray,[1,4] Sarah Rhodes,[2,4] Ross A Atkinson,[1,4] Katy Rothwell,[4] Paul Wilson,[3,4] Jo C Dumville,[1,4] Nicky A Cullum.[1,4,5]

Author Affiliations

1 Division of Nursing, Midwifery and Social Work, School of Health Sciences, Faculty of Biology, Medicine and Health, University of Manchester, Manchester, UK

2 Centre for Biostatistics, Faculty of Biology, Medicine and Health, University of Manchester, Manchester, UK

3 Alliance Manchester Business School, University of Manchester, Manchester, UK

4 NIHR CLAHRC Greater Manchester, Salford Royal NHS Foundation Trust, Salford, Manchester, UK

5 Research and Innovation Division, Manchester University NHS Foundation Trust, Grafton Street, Manchester, UK

Word Count

Abstract: 286; Manuscript: 4032

For peer review only

Key Words

Health services research, Evidence based practice, Wound management, Healthcare value, Healthcare quality, Prevalence survey, Patient-centred care,

Abstract

Background

Complex wounds impose a substantial health economic burden worldwide. As wound care is managed across multiple settings by a range of healthcare professionals with varying levels of expertise, the actual care delivered can vary considerably and result in the underuse of evidence-based interventions, the overuse of interventions supported by limited evidence and low value healthcare.

Objectives

To quantify the number, type and management of complex wounds being treated over a two-week period of time and to explore variations in care by comparing current practices in wound assessment, prevention and treatment.

Design

A multi-service cross sectional survey.

Setting

This survey spanned eight community services within five Northern England NHS Trusts.

Results

The point prevalence of complex wounds in this community based population was 16.4 per 10,000 (95% confidence interval 15.9 to 17.0). Based on data from 3179 patients, antimicrobial dressings were being used as the primary dressing for 36% of patients with complex wounds. Forty percent of people with leg ulcers either had not received the recommended Doppler-aided ABPI assessment, or it was unclear whether a

For peer review only

recording had been taken. Thirty-one percent of patients whose most severe wound was a venous leg ulcer were not receiving compression therapy and there was limited use of two-layer compression hosiery. Of patients with a pressure ulcer, 39% were not using a pressure relieving cushion or mattress.

Conclusions

Marked variations were found in care, underuse of evidence-based practices and overuse of practices that are not supported by robust research evidence. Significant opportunities for delivering better value wound care therefore exist. Efforts should now focus on developing strategies to identify, assess and disinvest from products and practices supported by little or no evidence, and enhance the uptake of those that are.

Article Summary

Strengths and limitations of this study

- This cross sectional survey provides robust community-focused population point prevalence estimates for different types of complex wounds.
- This is the first community-focused multi-service survey to capture the wide variation in treatment and care of complex wounds between different NHS trusts.
- The survey is based on wound care provided by community services and may have missed patients only treated by other services providers such as acute or primary care or those self-treating.
- As this was an anonymised survey we were unable to neither conduct any case validation nor validate wound aetiology.

For peer review only

For peer review only - http://bmjopen.bmj.com/site/about/guidelines.xhtml

Introduction

Complex wounds, (wounds with superficial, partial or full thickness skin loss healing by secondary intention) such as lower limb diabetic or venous ulcers, pressure ulcers, open trauma and surgical wounds,[1 2] impose a substantial health economic burden worldwide. In the UK, the point prevalence of complex wounds is estimated at 14.7 per 10,000 population, suggesting that approximately 80,000 people in the UK have one or more complex wound at any one time.[3] Whilst the annual cost of managing wounds has been crudely estimated at £3 billion in the UK,[4] $2.85 billion in Australia[5] and $25 billion in the US,[6] the true cost is unknown. The increasing prevalence of complex wounds with age[7] and multimorbidity[8] means that it is difficult to separate the cost of wound care from the cost of caring for people with complex needs.

Wound care is managed across multiple settings by a range of healthcare professionals (HCPs) with varying levels of expertise.[9-13] HCPs are constantly under pressure to make the right choices for their patients, but when faced with an array of wound care products to choose from,[14] inaccessible or limited research evidence to guide decisions[9-14] and silo-based cost-control measures,[15] decisions may not be based on best practice[16 17] and fragmentation of practice and services may occur.[18] With such diversity, it is unlikely that all patients with wounds will have access to good value healthcare.[19] Unwarranted variation in healthcare at a time of rising demand is a concern for health systems globally. It has been estimated that around a third of medical practices are effective or likely to be effective, 50% are of unknown effectiveness and 15% are harmful or unlikely to be beneficial.[20] The cost-effectiveness[21] and value are even less well known.

Value in healthcare is centred on the interests and activities of all stakeholders and is measured by outcomes achieved relative to cost. This perspective shifts the focus on the delivery of care from volume alone to the value gained for patients from the healthcare investments being made e.g. staff time and treatment costs.[19] Every individual should have access to value-based care that considers their needs, preferences and priorities.[19] Gaining maximum healthcare value, given available resources, has been central to discussions and policy changes worldwide in recent years.[22] Decision makers, especially those at policy and organisational levels, are increasingly aiming to maximise patient benefit whilst minimising the opportunity

For peer review only

costs of current healthcare approaches. The term "opportunity cost" refers to the potential for utilised resources to achieve more value elsewhere in the healthcare system.[23] High opportunity costs occur with the overuse of ineffective treatments (or those that make a very small clinical difference), leading to wasted patient benefit and reduced value in the healthcare system. Conversely, underuse of treatments known to be effective also leads to waste.[22]

In some cases, large improvements in the value of healthcare can be made relatively easily by the implementation of evidence based guidelines[14 24 25] and smarter procurement of services and products.[26] We are seeing the development of initiatives focussed on reducing variation and improving value, not only in the UK,[23 26-28] but across the world.[29-31] One campaign launched in 2012, 'Choosing Wisely', has been adopted by 12 countries.[32]

The first step to reducing unwarranted variation is to identify: 1) overuse of interventions that do not clearly offer value and 2) underuse of interventions known to offer value. Once such situations have been identified, organisations can start to work towards increased use of effective practices across the relevant populations they are responsible for. To date there have been no such initiatives focused in wound care; a significant yet often neglected area of care and healthcare spend, where there is great potential for better value care. To this end we explored variation in common interventions for complex wounds across eight community services (spanning five NHS Trusts) in the North of England. This project forms part of a wider wound care programme developed by the National Institute for Health Research Collaboration for Leadership in Applied Health Research and Care Greater Manchester (NIHR CLAHRC GM). Given the dearth of basic epidemiological data on chronic or complex wounds in community settings,[33-35] we sought to quantify the number, type and management of complex wounds being treated over a two-week period. We then compared current practices in wound assessment, prevention and treatment with evidence-based recommendations, allowing exploration of variations in care between areas where distinct healthcare organisations provide care to geographically proximal residents. Key treatments of interest included the use of antimicrobial wound dressings, compression for people with venous leg ulcers (VLUs) and the use of

For peer review only

For peer review only - http://bmjopen.bmj.com/site/about/guidelines.xhtml

pressure relief for pressure ulcer prevention, as these are specifically highlighted in recent guidelines;[14 24 36 37 38] as presented in Box 1.

Box 1. Key evidence and recommendations related to the assessment, prevention and treatment of complex wounds

Wound	Key treatments of interest	Guidelines and Recommendations
**Infected (All complex wounds)**	Silver dressings	Insufficient evidence to support the use of silver-containing dressings to promote wound healing or prevent wound infections.[14]
	Honey	Some high quality evidence (based on two randomised controlled trials (RCTs) only) has shown honey to heal partial thickness burns and infected post-operative wounds more quickly than comparators, however, comparators may not be relevant to current practice. Insufficient evidence to support the use of honey in other wounds.[14]
	Iodine	There is insufficient evidence addressing effectiveness and safety for use of iodine to treat or prevent wound infection.[14]
**Venous leg ulcer (VLU)**	Ankle Brachial Pressure Index (ABPI)	Measurement of ABPI should be performed by appropriately trained practitioners to substantiate the presence or absence of peripheral arterial disease (PAD) at initial assessment and to regularly review the use of compression therapy[37]
	Compression therapy	Simple non-adherent dressings and high compression multicomponent bandaging should be used for treating VLU patients with ABPI ≥ 0.8. Graduated compression hosiery is recommended to prevent recurrence of VLU.[37]
		Two layer compression stockings are as clinically effective as high compression bandages but more cost effective[35]
	Pentoxifylline	High quality evidence based on systematic review and meta-analysis has found improved VLU healing with the use of pentoxifylline (believed to increase microcirculatory blood flow although exact mechanism of action is unknown) and should be considered in patients with VLU.[37 39]
**Diabetic foot ulcer**	Dressing choice	Insufficient evidence to support the use of any specific dressing. Clinical assessment and patient preference should be taken into consideration whilst the lowest acquisition cost appropriate to the clinical circumstances should be used.[14 36]
	Pressure relief	Offer non-removable casting to offload plantar neuropathic, non-ischaemic, uninfected forefoot and midfoot diabetic ulcers taking into consideration clinical assessment and patient preference. Use pressure redistributing devices and strategies to minimise the risk of pressure ulcers developing.[36]
**Pressure ulcer (PU)**	Dressing choice	Insufficient evidence to support the use of any specific dressing, choice should be determined by the patient's pain, tolerance, location of the ulcer and amount of exudate. A dressing that promotes a warm, moist wound healing environment should be considered for grade 2, 3 and 4 Pus.[14 24]
	Pressure relief	Use high-specification foam mattresses, or consider the use of dynamic support surface if not sufficient. Consider high specification foam or equivalent pressure redistributing cushion for chair or wheelchair use.[24]

For peer review only - http://bmjopen.bmj.com/site/about/guidelines.xhtml

Materials and Methods

Project design and participating organisations

A multi-service, cross sectional survey recorded wound prevalence and care for people living with complex wounds across eight community services within five NHS Trusts in the north of England (with a population of 1.9 million). The methods used were based on a previous multi-service complex wound survey undertaken in Leeds, UK.[3]

As most wound care takes place in the community, the project focused on community-based wound care and did not include hospitals or primary care. HCPs working in a range of services (including tissue viability, adult and children's community nursing, podiatry, intermediate care, burns and plastics, specialist leg ulcer and specialist diabetic foot teams,) collected data for consecutive patients over a period of two weeks at each site between June 2015 and September 2016. Face-to-face training and accompanying instructions were provided to improve data accuracy and conformity of responses. Reminders were provided via telephone and email in the week leading to commencement. All services had a helpline number they could call if they had any questions either before or during the process.

Data collection

A structured questionnaire was developed (Appendix 1), largely based on one used successfully in a similar study.[3] The questionnaire was designed to capture the number and nature of wounds and the care provided in terms of workforce, service configuration, assessments used (i.e. ankle brachial pressure index (ABPI)), treatment choices, and product usage. Survey forms were delivered to all clinical areas across the five community NHS Trusts. Data were collected for all patients receiving one or more episodes of wound care for their complex wound(s) from an NHS community service during a two-week period. Forms were completed following the consultation during office time, without the patient present and were anonymised at source. To reduce duplication, forms were completed by the person providing the most hands-on care. This was supported by further local processes, such as placing stickers in patients' clinical notes when a form

For peer review only

had been completed. Potential duplicates were not removed from the dataset; as data were anonymised we could not be sure whether they were true duplications. This survey was defined as service review by the Trusts' Research and Development Departments and did not require approval from a Research Ethics Committee.

The survey consisted of structured questions relating to patient demographics, wound identification by type, number and severity. The form also asked for more detail about each patient's most severe wound. Thus if a patent had multiple complex wounds, more detailed information (e.g. on wound treatment) was only collected for one wound. For patients with multiple wounds of equal severity, HCPs were asked to focus on the largest wound. Information requested relating to investigations undertaken (e.g. Doppler-aided measurement of ABPI) or treatments provided (e.g. providing pressure relieving equipment) are all specific aspects of care carried out in community settings by the HCPs (and their colleagues) responsible for completing the questionnaire, and it would be expected that such clinical procedures would be recorded in the patient's community health record. The questionnaire had been used previously[3] and was piloted locally prior to commencement of the survey by a range of HCPs. Minor changes were made in the light of feedback received.

Data Analysis

All data were analysed using simple summary statistics; numbers with percentages for categorical data and mean/median values with range for numerical data. Community point prevalence rates per 10,000 population were produced for each wound type, along with 95% confidence intervals (CI). The denominator used for these calculations was 1 935 683 based on total population figures for the five trusts surveyed, taken from Health and Social Care Information Centre 2015 data.

For peer review only - http://bmjopen.bmj.com/site/about/guidelines.xhtml

Results

Description of community complex wound population

Overall we recorded data for 3179 patients with a total of 5632 complex wounds (median number of wounds per patient 1, range 1-24), corresponding to an overall community point prevalence (CPP) of 16.4 per 10,000 (95% CI 15.9 to 17.0). People with complex wounds tended to be elderly (median age 74 years) with at least one comorbidity (median 1.0, range 0-9). Cardiovascular disease was the most frequently reported comorbidity (in 1808 patients; 57%) followed by diabetes (817; 26%) and arthritis (641; 20%). Just under a half of the patients with complex wounds were immobile or walked with difficultly (Table 1) and 494 (31%) of 1613 patients reported as being fully mobile were receiving home visits from a HCP. VLUs were the most prevalent complex wound type (n=612; CPP 3.2 per 10,000; 95% CI 2.9 to 3.4), followed by diabetic foot ulcers (n=488; CPP 2.5 per 10,000; 95% CI 2.3 to 2.7), traumatic wounds (n=428; CPP 2.2 per 10,000; 95% CI 2.0 to 2.4) and pressure ulcers (n=348; CPP 1.8 per 10,000; 95% CI 1.6 to 2.0).

For peer review only

196 **Table 1. Demographic characteristics of patients with at least one complex wound**

Characteristic	
Gender (n=2967)	
Male n (%)	1439 (49)
Female n (%)	1528 (51)
Ethnicity (n=3152)	
White British n (%)	2819 (89)
Other n (%)	336 (11)
Age(n=3120)	
Median (range)	74 (1-107)
**Accommodation (n=3157)**	
Own/rented home n (%)	2728 (86)
Nursing/residential home n (%)	348 (11)
Other n (%)	84 (3)
Number of comorbidities (n=3179)	
Median (range)	1 (0-9)
Continence (n=3029)	
No incontinence n (%)	2487 (82)
Urinary or faecal incontinence or both n (%)	542 (18)
Mobility (n=3141)	
Fully mobile n (%)	1613 (51)
Walks with difficulty n (%)	1091 (35)
Immobile n (%)	437 (14)
**Community point prevalence per 10,000 population for most common wound types n (CPP; 95% CI)**	
Venous leg ulcer	612 (3.2; 2.9 to 3.4)
Diabetic foot ulcer	488 (2.5; 2.3 to 2.7)
Traumatic wound	428 (2.2; 2.0 to 2.4)
Pressure ulcer	348 (1.8; 1.6 to 2.0)

197

198 *Use of wound dressings for all complex wounds*

199 Wound dressings are applied to all types of complex wound. Of the 3038 patients for whom data on the
200 ‘most severe wound’ were provided, 1096 (36%) patients were receiving an antimicrobial primary dressing:
201 383 (13%) a silver dressing and 713 (23%) patients were receiving other antimicrobial dressings such as
202 iodine or honey. There was marked variation in the use of antimicrobial dressings across community services
203 as shown in Figure 1; ranging from 18% of patients in one area to 69% in another. As noted in Box 1, there is
204 currently insufficient evidence to support the use of antimicrobial dressings to promote wound healing.[14] It is
205 highly uncertain that antimicrobial dressings are clinically or cost effective and there no high quality
206 evidence that they improve wound healing, reduce infection rates or reduce the prescribing of systemic
207 antibiotics.[14 40] For VLUs, the use of anti-microbial dressings is specifically not recommended.[37]

208

For peer review only - http://bmjopen.bmj.com/site/about/guidelines.xhtml

The assessment and treatment of leg ulcers

A leg ulcer was reported as being the most severe wound in 25% (n=770) of patients with a most severe wound selected. For these patients a Doppler-aided ABPI measurement is a crucial part of the assessment process to rule out significant peripheral arterial disease and determine treatment choice and access (Box 1).[37] In total, of those patients with one or more leg ulcer, 19% (n=150) did not have an ABPI recorded in community-held notes. The frequency of ABPI recording varied across services from 10% to 28% and for a further 21% (n=167) this information was either unknown or not reported (ranging from 13% to 22%).

A VLU was reported as being the most severe wound in 570 patients of whom 175 (31%) were recorded as receiving no compression therapy; ranging from 2% to 30% (compression is an effective first-line treatment for venous ulcers[41 42]). Half (n=287; 50%) of those with VLUs were managed with compression bandages, 79 (14%) with compression hosiery and 29 (5%) with a combination of the two (Figure 2). There was limited use of two layer compression hosiery across all areas surveyed despite its known cost-effectiveness relative to compression bandaging.[35] None of the patients with a diagnosis of VLU were prescribed pentoxifylline; a treatment shown to be clinically and cost effective and recommended to promote the healing of VLUs.[37]

Use of support surfaces in those at risk of pressure ulceration

Over one third (35%) of patients with complex wounds were reported as being at risk of pressure ulceration and 348 (11%) had a pressure ulcer at the time of survey. Of the 281 patients whose most severe wound was a pressure ulcer (who are thus at known high risk of further ulceration and should be receiving pressure relief[24]), 109 (39%) patients were reported as not having a pressure relieving cushion or mattress (ranging from 27% in one area to 64% in another; Figure 3). For the 711 patients with a foot ulcer selected as the most severe wound, 40% (n=286) were not receiving any pressure relief for the affected foot (ranging from 31% to 60%).

For peer review only - http://bmjopen.bmj.com/site/about/guidelines.xhtml

Discussion

This study characterised the number and nature of complex wounds being cared for by NHS community services, and the assessments and treatments being used in their management. Our estimate of the point prevalence of complex wounds (16.4 cases per 10,000 population) is consistent with another estimate from the North of England (14.7 per 10,000 population[3]). As these are point prevalence estimates from general populations using census figures as denominators, the estimates are lower than those estimates which focus on specific high-risk populations (such as hospital patients).[1] We found indicators that revealed unwarranted variation in clinical practice across participating services, the underuse of evidence-based interventions (e.g., compression therapy for venous ulcers) and the overuse of interventions supported by limited evidence (e.g., antimicrobial dressings). Overuse of such treatments could incur opportunity cost; drawing resource from the system that cannot be used to confer benefit elsewhere.

Overuse of interventions supported by limited evidence

Across community services, the survey revealed substantial variation in the use of antimicrobial dressings for wound management, ranging from 18% to 69% of all primary dressings used. The unit cost of antimicrobial dressings is often higher than that of their non-antimicrobial counterparts; their use is associated with a high cost for little or no known patient benefit. Whilst one could postulate that use of anti-microbial dressings might reduce the prescribing of systemic antibiotics (which would be highly desirable in the context of antimicrobial resistance[43]), there is no evidence that this is the case. To examine the use of anti-microbial dressings in more depth we considered silver dressings for which current evidence on the relative effectiveness for preventing or treating wound infection and promoting healing is uncertain.[44 45] Silver dressings cost several times more than their non-silver counterparts. In a conservative scenario where silver dressings are used for a patient over a 2-week period with two dressing changes per week, the dressing cost (using an average cost for a small dressing) would be approximately £32. The cost for a standard foam dressing (of the same size) in the same scenario is approximately five times less (£6.80). Use of silver dressings in this situation (assuming all other aspects of care and outcome are equal) corresponds to an

For peer review only - http://bmjopen.bmj.com/site/about/guidelines.xhtml

additional £2,520 per 100 patients treated with silver dressings. Tackling overuse is not an easy task and not solely the responsibility of individual HCPs. But despite increased awareness among clinicians, policy makers and the general public during the last 5-10 years, there have not been widespread changes in policy to address this issue.[46] In wound care specifically, removing silver dressings from a prescribing formulary would offer a partial solution only. There is a clear need for a rational strategy for identifying, assessing and disinvesting from products supported by little or no evidence; ideally this should be delivered at a health system level.[47]

Underuse of evidence-based interventions

Across our population we saw underuse of ABPI in those with leg ulcers (with 19% not having this measure recorded and for 21% it was unclear whether a recording had been taken). Such under-assessment (whether a delay or omission) is likely linked to the underuse of compression therapy also observed. These related issues require urgent attention since compression is a highly efficient and cost-effective treatment for VLU.[37] The first response to this finding is to seek an understanding of the reasoning behind the observed patterns of compression use. For example, underuse may be due to patients waiting for ABPI assessment, or ABPI assessment identifying peripheral arterial disease that may preclude compression use; in other cases, lack of compression may reflect issues with patient adherence to therapy or a shortage of staff with compression bandaging skills. The extent to which current findings can be explained by appropriate contraindications is unclear and requires further investigation. Crucially the reason for such wide variation between relatively local areas also needs further explanation.

Using data from RCTs,[48 49]we conservatively calculate that non-use of compression in those with VLUs will lead to 23 fewer people per 100 patients with VLU healing over 12 months compared with use of compression. Using an estimated mean cost per VLU episode of £1800[35] we can estimate an annual increased cost of £41,400 per 100 VLU patients not using compression. However, we could tentatively also consider non-use of compression as a proxy for sub-optimal care. If we apply the recent Rightcare[28]

For peer review only - http://bmjopen.bmj.com/site/about/guidelines.xhtml

estimated cost savings associated with optimised VLU care (including high compression use), savings could be as high as £118,979 per 100 VLU patients. While here we have focused on cost impacts, it is important to consider the improved health-related quality of life associated with a healed wound as well as the reduced financial burden on patients, especially those who are unable to work or require time away from work to attend clinic appointments.

There is also an apparent underuse of pentoxifylline, a xanthine derivative used to treat muscle pain in people with peripheral arterial disease, but also believed to increase microcirculatory blood flow in patients with VLU. Uptake is low despite evidence from 12 RCTs included in a systematic review[39] that it increases ulcer healing both with and without compression. The reasons for this underuse are being explored further but are likely to include: lack of awareness of the drug and its effects amongst both community GPs and nurses; relative lack of nurse prescribers able to prescribe pentoxifylline and reluctance to prescribe for an off-licence indication (particularly in people who may be taking several other medicines for comorbidities). It is also worth noting that the drug is low cost but not marketed because the patent is long expired. It is unclear whether this treatment is used more widely outside the UK. Using published relative effectiveness estimates and related costs,[50] we have estimated that use of pentoxifylline alongside compression could result in cost savings of over £40,000 per 100 VLU patients treated. Combined increases in the use of ABPI, cost-effective compression therapy and greater pentoxifylline use in the treatment of those with venous leg ulceration is likely to see a cumulative increase in improved patient outcomes, experiences and reduced health care costs.

Similar exploration is required in relation to the potential underuse of pressure relieving equipment suggested by our study, in order to understand the true scope for improvement. In all cases, further work is required to identify the factors that underpin clinical decision making and behaviour in these areas. An understanding of such factors would support the identification and selection of appropriate behaviour change techniques and implementation strategies, targeted at modifying these behaviours and generating improvement and thus value in healthcare. Having a pressure ulcer is prognostic for further pressure

For peer review only - http://bmjopen.bmj.com/site/about/guidelines.xhtml

ulceration, thus those with an ulcer are considered at risk and should receive pressure relieving interventions such as support surfaces. Even accounting for the initial cost of purchasing a device given the large cost associated with healing a pressure ulcer (estimated cost for healing a Grade 2 pressure ulcer = £5,241),[51] we conservatively calculate that the use of support surfaces to prevent pressure ulceration could save at least £38,000 per 100 at-risk patients. Again those with a pressure ulcer are also known to have worse health-related quality of life than others with similar co-morbidities.[52] These are not isolated examples of underuse: a US study found that patients with acute or chronic conditions (that represented the leading causes of illness, death and utilization of healthcare) received only 55% of recommended care[53] and similarly the CareTrack study found that Australians received appropriate care in only 57% of 35,573 eligible healthcare encounters.[54] However, this is the first work that has revealed so clearly, variation in the use of interventions for VLUs where there are corresponding guideline-based recommendations. Failure to deliver best practice is often a result of poor execution or lack of widespread adoption of best care processes.[55] International interest in research translation and quality improvement reflects the growing recognition of the slow and inconsistent uptake of effective healthcare practices worldwide.[56]

Disinvestment and implementation of improvement initiatives to promote better value care

Local variation in product choice between organisations is shown clearly in the work presented. This highlights the huge scope for better value care in both the assessment and treatment of complex wounds; value could be released by disinvestment in some areas with savings being focused on areas of underuse identified here. Further work should be undertaken to understand the factors that underpin decision making around treatment use, with a particular focus on exploring the motivations to use expensive treatments with limited evidence at a time when the NHS faces significant resource constraints. Working with our service partners, we plan to address the observed local practice variations through a programme of improvement work undertaken as part of NIHR CLAHRC GM. However, we recognise that this process needs to be replicated across the health system as a whole if overuse and underuse are to be addressed fully. As we have stated above, there is a clear need for a national strategy for identifying, assessing and disinvesting from

For peer review only

For peer review only - http://bmjopen.bmj.com/site/about/guidelines.xhtml

products and practices supported by little or no evidence.[47] A model for taking a systematic approach to disinvestment already exists in the form of the SHARE programme in Australia. This model enables clinicians, managers and policy makers to manage the process from identifying the need for disinvestment to implementing the change and evaluating outcomes.[57] Similar efforts are required in the UK if practice variations are to be addressed.

Strengths and limitations

This cross sectional survey provides robust community-focused population point prevalence estimates for different types of complex wounds and is the first multi-service survey to capture the wide variation in treatment and care of complex wounds between different NHS Trusts. There are a few limitations to our study. Firstly, the study looked specifically at patients receiving care from NHS community services and did not include people whose wound care was delivered by hospitals, primary care, or other care providers, and also those who may have been self-caring at this time. While this prevents us from comparing the characteristics of people with and without complex wounds at the time of the survey, collection of these data would have been too resource intensive for participating services and we know from previous work that the vast majority of people with wounds are cared for in community settings.[1] Secondly, we only scrutinised the patient information readily available to community staff and did not examine wider (e.g., GP) patient records. However given that we were looking for information regarded as crucial to the management of people with complex wounds, any omission of information from community records risks suboptimal clinical decision making. Thirdly, it is also conceivable that services failed to complete a survey form for each patient they saw with a complex wound during the survey period. However, both the level of engagement from services and the similarity between the estimated CPP obtained here and that found in a previous but smaller survey in Leeds,[3] suggest the impact of this is likely to be negligible. Finally, as this was an anonymised survey we were unable to neither conduct any case validation nor validate wound aetiology. Consequently, data presented reflects the treating HCP's assessment.

For peer review only - http://bmjopen.bmj.com/site/about/guidelines.xhtml

Conclusions

This survey adds important robust epidemiological data to the complex wound literature where existing prevalence data have been found to be limited when systematically reviewed.[1] We also highlight the over- and underuse of services and treatments and reflect the issues emphasised in a number of reports relating to Better Value Healthcare.[19 26 29] These findings suggest significant opportunities for delivering better value wound care exist. Efforts should now focus on developing strategies to identify, assess and disinvest from products and practices supported by little or no evidence and enhance the uptake of those that are.

Figure Legends

Figure 1. Proportion of complex wounds for which primary dressing contained antimicrobials: the other anti-microbial dressing group maps to the same section of the British National Formulary and includes dressings such as Polyhexanide polyhexamethylene biguanide (bars represent included community services), *Number of patients per community service ranged from 172 to 655*

Figure 2. Highest level of compression used for patients with venous leg ulcers selected as most severe wound (bars represent included community services). *Number of patients per community service ranged from 14 to 151*

Figure 3. Proportion of patients using pressure relieving mattresses or cushions (bars represent included community services). *Number of patients per community service ranged from 10 to 63*

References

1. Cullum N, Buckley H, Dumville J, et al. Wounds Research for Patient Benefit: a 5 year programme of research. *Programme Grants Appl Res* 2016;4(13)
2. Vowden K. Complex wound or complex patient. *internursecom* 2005;10(Sup2):S6-14.
3. Hall J, Buckley HL, Lamb KA, et al. Point prevalence of complex wounds in a defined United Kingdom population. *Wound Repair Regen* 2014;22(6):694-700. doi: 10.1111/wrr.12230 [published Online First: 2014/09/17]
4. Guest JF, Ayoub N, McIlwraith T, et al. Health economic burden that wounds impose on the National Health Service in the UK. *BMJ Open* 2015;5(12):e009283. doi: 10.1136/bmjopen-2015-009283
5. Graves N, Zheng H. Modelling the direct health care costs of chronic wounds in Australia. *Wound Practice and Research* 2014; 22(1).
6. Sen CK, Gordillo GM, Roy S, et al. Human skin wounds: a major and snowballing threat to public health and the economy. *Wound Repair Regen* 2009;17(6):763-71. doi: 10.1111/j.1524-475X.2009.00543.x
7. WHO. Global Health and Aging: National Institute on Aging, National Institutes of Heath, US Department of Health and Human Services, World Health Organisation; 2011 [Available from: http://www.who.int/ageing/publications/global_health.pdf.
8. Gould L, Abadir P, Brem H, et al. Chronic wound repair and healing in older adults: current status and future research. *J Am Geriatr Soc* 2015;63(3):427-38. doi: 10.1111/jgs.13332
9. Corbett LQ. Wound Care Nursing: Professional Issues and Opportunities. *Advances in Wound Care* 2011;1(5):189-93.

For peer review only - http://bmjopen.bmj.com/site/about/guidelines.xhtml

10. Harrison MB, Graham ID, Lorimer K, et al. Nurse clinic versus home delivery of evidence-based community leg ulcer care: a randomized health services trial. *BMC Health Serv Res* 2008;8:243. doi: 10.1186/1472-6963-8-243

11. Medical Advisory Secretariat. Community-based care for chronic wound management: an evidence-based analysis. *Ontario Health Technology Assessment Series* 2009;9(18)

12. Moore Z, Butcher G, Corbett LQ, et al. AAWC, AWMA, EWMA Position Paper: Managing Wounds as a Team *J Wound Care* 2014;23:S1-S38.

13. Whitlock E, Morcom J, Spurling G, et al. Wound care costs in general practice - a cross-sectional study. *Aust Fam Physician* 2014;43(3):143-6.

14. NICE. Chronic wounds: advanced wound dressings and antimicrobial dressings: Evidence summary [ESMPB2]: National Institute for Health and Care Excellence; 2016 [Available from: https://www.nice.org.uk/guidance/esmpb2/resources/chronic-wounds-advanced-wound-dressings-and-antimicrobial-dressings-1502609570376901 (accessed November 2017).

15. Erstad BL. Value-Based Medicine: Dollars and Sense. *Crit Care Med* 2016;44(2):375-80. doi: 10.1097/CCM.0000000000001559

16. Gabbay J, le May A. Evidence based guidelines or collectively constructed "mindlines?" Ethnographic study of knowledge management in primary care. *BMJ* 2004;329(7473):1013. doi: 10.1136/bmj.329.7473.1013

17. Thompson C, Cullum N, McCaughan D, et al. Nurses, information use, and clinical decision making--the real world potential for evidence-based decisions in nursing. *Evid Based Nurs* 2004;7(3):68-72.

18. Enthoven AC. Integrated delivery systems: the cure for fragmentation. *Am J Manag Care* 2009;15(10 Suppl):S284-90.

19. Porter ME. What is value in health care? *N Engl J Med* 2010;363(26):2477-81. doi: 10.1056/NEJMp1011024

20. Prasad V, Vandross A, Toomey C, et al. A decade of reversal: an analysis of 146 contradicted medical practices. *Mayo Clin Proc* 2013;88(8):790-8. doi: 10.1016/j.mayocp.2013.05.012

21. Productivity Commission. Impacts of Advances in Medical Technology in Australia. Research Report 2005 [Available from: http://www.pc.gov.au/inquiries/completed/medical-technology. (accessed May 2017) accessed May 2017 2017.

22. WHO. Appropriateness in Health Care Services. In: WHO Regional Office for Europe, ed. Copenhagen, Denmark World Health Organisation, 2000.

23. Muir Gray JA. How to Get Better Value Healthcare Second ed. Oxford, UK: Offox Press for Better Value Healthcare Ltd 2011.

24. NICE. Pressure ulcers: prevention and management Clinical guideline [CG179]: National Institute for Health and Care Excellence, 2014.

25. Ubbink DT, Santema TB, Stoekenbroek RM. Systemic wound care: a meta-review of cochrane systematic reviews. *Surg Technol Int* 2014;24:99-111.

26. Alderwick H, Robertson R, Appleby J, et al. Better value in the NHS: The role of changes in clinical practice. London, UK: The King's Fund, 2015.

27. Cripps M. NHS Right Care – expanding the approach in the context of delivering the Five Year Forward View: NHS England; 2015 [Available from: https://www.england.nhs.uk/wp-content/uploads/2015/12/nqb-oct15-5.pdf (accessed April 2017) accessed 24th April 2017.

28. NHS RightCare. NHS RightCare scenario: The variation between sub-optimal and optimal pathways. Betty's story: Leg ulcer wound care NHS England; 2017 [Available from: https://www.england.nhs.uk/rightcare/wp-content/uploads/sites/40/2017/01/nhs-rightcare-bettys-story-narrative-full.pdf (accessed May 2017).

29. Levinson W, Kallewaard M, Bhatia RS, et al. 'Choosing Wisely': a growing international campaign. *BMJ Qual Saf* 2015;24(2):167-74. doi: 10.1136/bmjqs-2014-003821

30. ABIM Foundation. Choosing Wisely Philadelphia: ; 2017 [Available from: http://www.choosingwisely.org/ (accessed June 2017) accessed 18th May 2017 2017.

31. Canadian Medical Association. Choosing Wisley Canada University of Toronto 2017 [Available from: http://www.choosingwiselycanada.org/ (accessed May 2017).

32. Bhatia RS, Levinson W, Shortt S, et al. Measuring the effect of Choosing Wisely: an integrated framework to assess campaign impact on low-value care. *BMJ Qual Saf* 2015;24(8):523-31. doi: 10.1136/bmjqs-2015-004070

33. Hodgson R, Allen R, Broderick E, et al. Funding source and the quality of reports of chronic wounds trials: 2004 to 2011. *Trials* 2014;15:19. doi: 10.1186/1745-6215-15-19

34. Lazarus GS, Kirsner RS, Zenilman J, et al. Clinical interventions for venous leg ulcers: Proposals to improve the quality of clinical leg ulcer research. *Wound Repair Regen* 2016;24(5):767-74. doi: 10.1111/wrr.12466

35. Ashby RL, Gabe R, Ali S, et al. Clinical and cost-effectiveness of compression hosiery versus compression bandages in treatment of venous leg ulcers (Venous leg Ulcer Study IV, VenUS IV): a randomised controlled trial. *Lancet* 2014;383(9920):871-9. doi: 10.1016/S0140-6736(13)62368-5

36. NICE. Diabetic foot problems: prevention and management [NG19]: National Institute for Health and Care Excellence, 2015.

For peer review only

For peer review only - http://bmjopen.bmj.com/site/about/guidelines.xhtml

37. SIGN. Management of chronic venous leg ulcers: A national clinical quideline (120): Scottish Intercollegiate Guidelines Network, 2010.
38. Kelly J, Clifton E, Fearns N, Harbour J, Heller-Murphy S, Herbert P, Macpherson K, Myles S, Santo V, Wilson L Antimicrobial wound dressings (AWDs) for chronic wounds. Health Technology Assessment report 13: Health Improvement Scotland, 2015.
39. Jull AB, Arroll B, Parag V, et al. Pentoxifylline for treating venous leg ulcers. *Cochrane Database Syst Rev* 2012;12:CD001733. doi: 10.1002/14651858.CD001733.pub3
40. O'Meara S, Al-Kurdi D, Ologun Y, et al. Antibiotics and antiseptics for venous leg ulcers. *Cochrane Database Syst Rev* 2014(1):CD003557. doi: 10.1002/14651858.CD003557.pub5
41. Nelson EA, Bell-Syer SE. Compression for preventing recurrence of venous ulcers. *Cochrane Database Syst Rev* 2014(9):CD002303. doi: 10.1002/14651858.CD002303.pub3
42. O'Meara S, Cullum N, Nelson EA, et al. Compression for venous leg ulcers. *Cochrane Database Syst Rev* 2012;11:CD000265. doi: 10.1002/14651858.CD000265.pub3
43. WHO. Antibiotic Resistance: Multi-country public awareness survey: World Health Organization, 2015.
44. Storm-Versloot MN, Vos CG, Ubbink DT, et al. Topical silver for preventing wound infection. *Cochrane Database Syst Rev* 2010(3):CD006478. doi: 10.1002/14651858.CD006478.pub2
45. Vermeulen H, van Hattem JM, Storm-Versloot MN, et al. Topical silver for treating infected wounds. *Cochrane Database Syst Rev* 2007(1):CD005486. doi: 10.1002/14651858.CD005486.pub2
46. Brownlee S, Chalkidou K, Doust J, et al. Evidence for overuse of medical services around the world. *Lancet* 2017 doi: 10.1016/S0140-6736(16)32585-5
47. Prasad V, Ioannidis JP. Evidence-based de-implementation for contradicted, unproven, and aspiring healthcare practices. *Implement Sci* 2014;9:1. doi: 10.1186/1748-5908-9-1
48. Ashby RL, Gabe R, Ali S, et al. VenUS IV (Venous leg Ulcer Study IV) - compression hosiery compared with compression bandaging in the treatment of venous leg ulcers: a randomised controlled trial, mixed-treatment comparison and decision-analytic model. *Health Technol Assess* 2014;18(57):1-293, v-vi. doi: 10.3310/hta18570
49. Morrell CJ, King B, Brereton L. Community-based leg ulcer clinics: organisation and cost-effectiveness. *Nurs Times* 1998;94(9):51-4.
50. Iglesias CP, Birks Y, Nelson EA, et al. Quality of life of people with venous leg ulcers: a comparison of the discriminative and responsive characteristics of two generic and a disease specific instruments. *Qual Life Res* 2005;14(7):1705-18.
51. Bennett G, Dealey C, Posnett J. The cost of pressure ulcers in the UK. *Age Ageing* 2004;33(3):230-5. doi: 10.1093/ageing/afh086
52. Essex HN, Clark M, Sims J, et al. Health-related quality of life in hospital inpatients with pressure ulceration: assessment using generic health-related quality of life measures. *Wound Repair Regen* 2009;17(6):797-805. doi: 10.1111/j.1524-475X.2009.00544.x
53. McGlynn EA, Asch SM, Adams J, et al. The quality of health care delivered to adults in the United States. *N Engl J Med* 2003;348(26):2635-45. doi: 10.1056/NEJMsa022615
54. Runciman WB, Hunt TD, Hannaford NA, et al. CareTrack: assessing the appropriateness of health care delivery in Australia. *Med J Aust* 2012;197(2):100-5.
55. Berwick DM, Hackbarth AD. Eliminating waste in US health care. *JAMA* 2012;307(14):1513-6. doi: 10.1001/jama.2012.362
56. Glasziou P, Straus S, Brownlee S, et al. Evidence for underuse of effective medical services around the world. *Lancet* 2017 doi: 10.1016/S0140-6736(16)30946-1
57. Harris C, Green S, Ramsey W, et al. Sustainability in Health care by allocating resources effectively (SHARE) 1: introducing a series of papers reporting an investigation of disinvestment in a local healthcare setting. *BMC Health Serv Res* 2017;17(1):323. doi: 10.1186/s12913-017-2210-7

Funding

This project was funded by the National Institute for Health Research Collaboration for Leadership in Applied Health Research and Care (NIHR CLAHRC) Greater Manchester. The NIHR CLAHRC Greater Manchester is a

For peer review only

For peer review only - http://bmjopen.bmj.com/site/about/guidelines.xhtml

partnership between providers and commissioners from the NHS, industry and the third sector, as well as clinical and research staff from the University of Manchester. The views expressed in this article are those of the author(s) and not necessarily those of the NHS, NIHR or the Department of Health.

Contributors

NC and JD conceived the idea and design for the overall project, TG, KR and PW contributed to further development of the study design, TG and KR conducted stakeholder consultation with practitioners, SR was responsible for data analysis, RA and JD contributed to data analysis. TG created the original draft of the manuscript. All authors contributed to the interpretation of study findings, critical revision of the manuscript for important intellectual content and approval of the final manuscript.

Acknowledgements

The authors are grateful to the healthcare professionals, from NHS partner organisations, involved in data collection, for giving their time and enthusiastically supporting this project. The authors would also like to thank Wanda Russell (NIHR CLAHRC GM Research Fellow at the time of this project) for her role in conducting stakeholder consultation with practitioners and data collection and Louise Hussey, Research Fellow, for her contribution to data analysis.

Competing interests

None declared.

For peer review only

Data sharing statement

Requests for access to data should be addressed to the corresponding author.

For peer review only

For peer review only - http://bmjopen.bmj.com/site/about/guidelines.xhtml

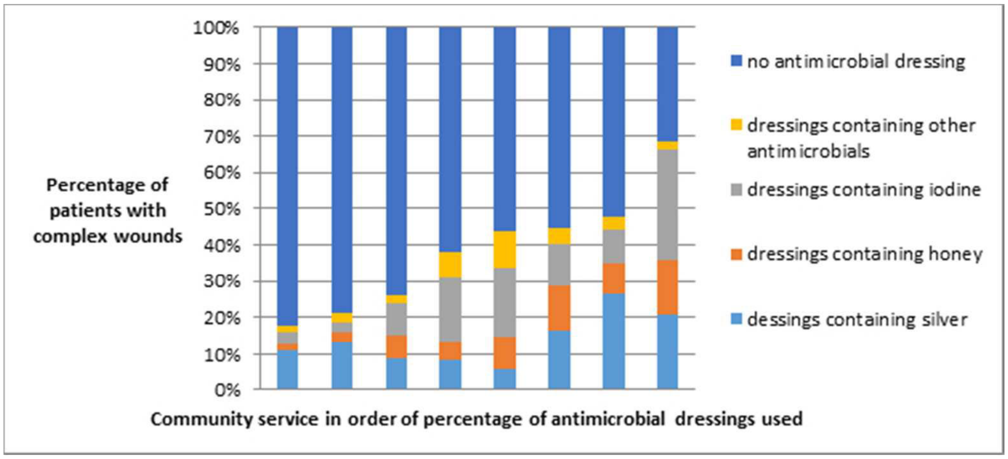

52x23mm (300 x 300 DPI)

For peer review only

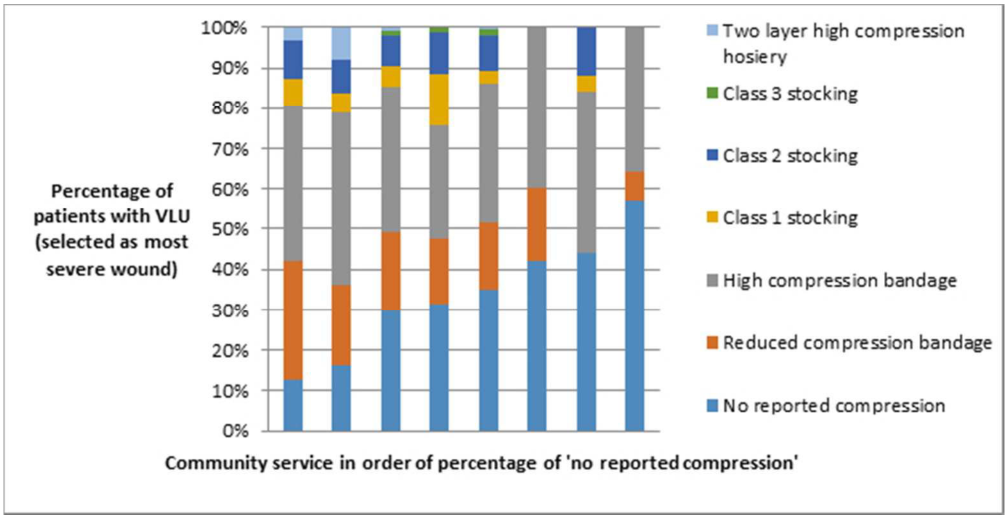

52x27mm (300 x 300 DPI)

For peer review only

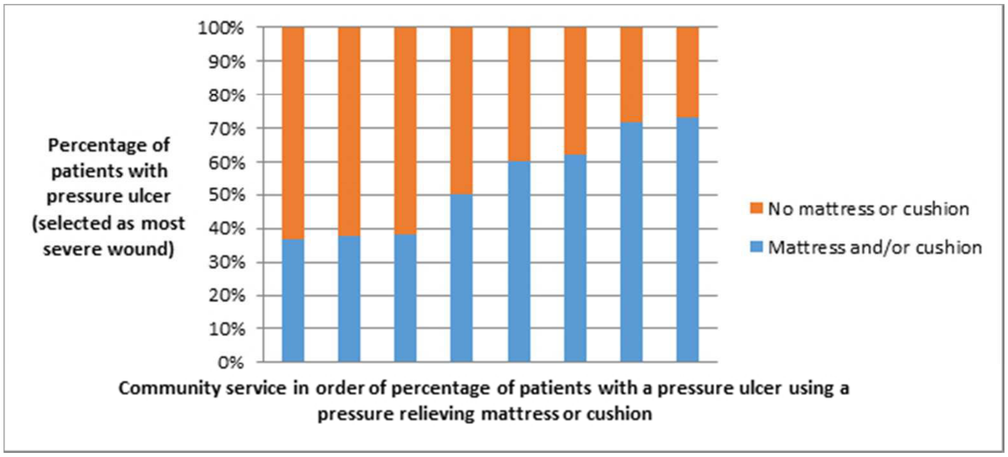

52x23mm (300 x 300 DPI)

Office use only: ID number

☐☐☐☐

The University of Manchester

National Institute for Health Research
Collaboration for Leadership in Applied Health Research and Care (CLAHRC) Greater Manchester

A Survey of Complex Wounds and their Care in the Community

Patient Data Capture Form

Date this form was completed: ☐☐ / ☐☐ / ☐☐☐☐
day month year

This form is to be completed by the NHS health care professional (from the participating NHS community-based services) delivering most of the hands on wound care for a person with one or more complex wounds. Please aim to ensure that only one form is completed per patient

For peer review only

Section 1:

1.1 What is your main clinical role currently? *(Please cross (x) one option)*

District Nurse/community nurse ☐ Nurse Specialist ☐

Podiatrist ☐ Other ☐

If 'Other' please state: ☐

1.2 What is your NHS Agenda for Change band (as the health care professional from the participating NHS community-based services delivering most of the hands on wound care for this patient)? *(Please cross (x) one option)*

Band 3 ☐ Band 4 ☐ Band 5 ☐ Band 6 ☐ Band 7 ☐ Band 8a ☐

Other ☐ If 'Other' please state: ☐

1.3 Currently, in which location does this patient receive most of their hands on wound care? *(Please cross (x) one option)*

Patient's home ☐ Community clinic/heath centre/GP practice ☐ Nursing home ☐

Residential home ☐ Hospice ☐ Prison ☐

Other ☐ If 'Other' please state: ☐

1.4 How many wound related consultations (with all professionals involved) has THIS patient had in the last seven days?: ☐☐

1.5 Duration of the most recent consultation: ☐☐☐ minutes

1.6 Are any other health professionals involved in deciding or planning wound care for this person? ***(Please cross (x) one option)***

Yes ☐ **No** ☐ Don't know ☐

↓

1.7 If Yes who are they? ***(please cross (x) all that apply)*****:**

GP ☐ Tissue viability nurse ☐ Other wound care specialist ☐

Podiatrist ☐ District nurse ☐

Other ☐ If 'Other' please state: ☐

For peer review only

Section 2: Patient details

2.1 Patient's DOB: dd ☐☐ mm ☐☐ yyyy ☐☐☐☐ **2.2 Patient's gender:** male ☐ female ☐

2.3 Ethnicity:

White British ☐ White Irish ☐ White Other ☐ Black African ☐ Black Caribbean ☐ Black Other ☐

Asian Indian ☐ Asian Pakistani ☐ Asian Bangladeshi ☐ Asian Other ☐ White and Black Caribbean ☐ White and Black African ☐

White and Asian ☐ Other mixed background ☐ Chinese ☐ Other ☐

2.4 Please state location of patient's GP ***(Please cross (x) one option)***

Central Manchester ☐ South Manchester ☐ Salford ☐ Oldham ☐

Bury ☐ Heywood, Middleton, Rochdale ☐ Trafford ☐

Other location ☐ If other, please state: ☐

Don't know ☐

2.5 What type of accommodation best describes where this patient currently lives? *(Please cross (x) one option)*

Own / rented home ☐ Nursing home ☐ Residential home ☐

No fixed abode ☐ Prison ☐

Other ☐ If 'Other' please state: ☐

2.6 What co-morbidities does this patient have? *(please cross (x) all that apply)*:

**Cardiovascular disease (CVD)**	e.g. Hypertension (high blood pressure, high BP), Myocardial infarction (MI, heart attack, IHD), Angina (IHD), Heart failure (CCF, CHF, HF, LVF)	☐
**Peripheral Arterial Disease**	e.g. Ischaemia (leg/foot), Claudication (intermittent claudication), Rest pain, Arteriopathy/angiopathy, Aneurysm including aortic aneurysm	☐
**Diabetes (DM)**	e.g. Insulin dependent DM (IDDM, Type I diabetes), Non insulin dependent DM (NIDDM, Type II diabetes)	☐
**Arthritis**	e.g. Rheumatoid arthritis (RA), Osteoarthritis (OA), Non rheumatoid arthritis	☐
**Orthopaedics**	e.g. Fractured neck of femur (# NOF, # hip, fractured hip, broken hip), Other fracture/break, Elective orthopaedic	☐
**Airways**	e.g. Chronic obstructive airways/pulmonary disease (COAD, COPD), Chronic bronchitis, Emphysema, Asthma	☐
**Neurological**	e.g. Parkinson's disease, Multiple sclerosis (MS), Epilepsy (fits, Grand mal, Petit mal, tonic-clonic, seizure), Spinal Injury	☐
**Stroke**	e.g. (cerebrovascular accident, CVA, transient ischaemic attack, TIA)	☐
**Cancer**	e.g. any cancer diagnosis	☐

For peer review only

2.7 Patient's Continence *(please cross (x) all that apply)*:

No incontinence ☐ Urinary incontinence ☐ Faecal Incontinence ☐

2.8 Patient mobility *(please cross (x) one option)*:

Patient walks freely ☐ Patient walks with difficultly ☐ Patient is immobile ☐

2.9 In your judgement is this patient at risk of pressure ulceration? *(please cross (x) one option):*

Yes ☐ No ☐ Don't know ☐

↓

2.10 If yes is their pressure ulcer risk documented in their notes/care plan? *(please cross (x) one option)*:

Yes ☐ No ☐ Don't know ☐

Section 3: Current complex wounds

3.1 Please record the number of wounds (by wound type) that this patient has.

Wound type:		Number of wound of this type (please circle one number for each type or leave blank if 0):							
Foot ulcer in patient with diabetes	RIGHT foot	1	2	3	4	5	6	7	8 or more
	LEFT foot	1	2	3	4	5	6	7	8 or more
Foot ulcer in patient without diabetes	RIGHT foot	1	2	3	4	5	6	7	8 or more
	LEFT foot	1	2	3	4	5	6	7	8 or more
Venous leg ulcer	RIGHT leg	1	2	3	4	5	6	7	8 or more
	LEFT leg	1	2	3	4	5	6	7	8 or more
Arterial leg ulcer	RIGHT leg	1	2	3	4	5	6	7	8 or more
	LEFT leg	1	2	3	4	5	6	7	8 or more
Arterial/venous (mixed aetiology) leg ulcer	RIGHT leg	1	2	3	4	5	6	7	8 or more
	LEFT leg	1	2	3	4	5	6	7	8 or more
Pressure ulcer		1	2	3	4	5	6	7	8 or more

For peer review only

**Dehisced surgical wound currently healing by secondary intention**	1	2	3	4	5	6	7	8 or more
**Wound resulting from excision of pilonidal sinus**	1	2	3	4	5	6	7	8 or more
**Wound resulting from excision of peri-anal abscess**	1	2	3	4	5	6	7	8 or more
**Other surgical wound healing by secondary intention**	1	2	3	4	5	6	7	8 or more
**Traumatic wound (e.g. result of road traffic accident)**	1	2	3	4	5	6	7	8 or more
**Fungating carcinoma**	1	2	3	4	5	6	7	8 or more
**Burn**	1	2	3	4	5	6	7	8 or more

Other wound type - please describe: **Number of wound of this type (please circle):**

	1	2	3	4	5	6	7	8 or more
	1	2	3	4	5	6	7	8 or more
	1	2	3	4	5	6	7	8 or more

For peer review only

Section 4: Treatment of complex wounds

We now want to collect information on the treatment of this patient's complex wound(s). If the patient has more than one complex wound (of any type) please now focus on the one wound you consider to be the worst/most severe (if there are more than one wound of equal severity choose any one of these wounds and complete the following information for just this wound).

We will refer to the wound for which the following data are collected as the selected wound.

4.1 Please record what type of wound the selected wound is (as noted above - this is the most severe/wound and therefore the one you are focusing on in this section). ***Please cross (x) one option.***

Foot ulcer in patient with diabetes ☐ Foot ulcer in patient without diabetes ☐

Venous leg ulcer ☐ Arterial leg ulcer ☐

Arterial/venous leg ulcer ☐ Pressure ulcer ☐

Dehisced surgical wound currently healing by secondary intention ☐ Wound resulting from excision of pilonidal sinus ☐

Wound resulting from excision of peri-anal abscess ☐ Other surgical wound healing by secondary intention ☐

Traumatic wound (e.g. result of road traffic accident) ☐ Fungating carcinoma ☐

Burn ☐

Other wound type ☐ If 'Other' please state: ☐

4.2 Please note the location/area most affected by the selected wound. ***Please cross (x) one option.***

head/neck ☐ arm ☐ hand ☐ breast ☐

abdomen ☐ back ☐ sacrum ☐ natal cleft ☐

buttock ☐ peri-anal ☐ perineum ☐ leg ☐

foot ☐ Other ☐ If 'Other' please state: ☐

Please record the following details for the selected wound. ***Please cross (x) one option.***

4.3 Exudate level:

High ☐ Moderate/ medium ☐ Low ☐ None ☐

4.4 Malodour:

Yes ☐ No ☐

For peer review only - http://bmjopen.bmj.com/site/about/guidelines.xhtml

4.5 For the selected wound, which wound dressings are being used currently? ***(Refer to the list of dressings in the guidelines and insert the corresponding code in each of the boxes)*****:**

Primary dressing [ ][ ] Secondary dressing [ ][ ]

Please record any other wound treatments for the selected wound:

4.6 Drugs/medicines: ***(please cross (x) all that apply)***

Antibiotics for wound infections [ ] Pentoxifylline [ ]

Topical steroids [ ] Analgesics [ ]

Other [ ] If 'Other' please state: [ ]

4.7 For the selected wound, the bandage currently used: (***Please cross (x) one option*****).**

N/A [ ] 4 layer compression bandage [ ]

Short stretch bandage (e.g. Actico/Comprilan) [ ] 3 layer reduced compression bandage [ ]

2 layer compression bandage (e.g. K2) [ ] Non-compression bandage (plus wadding) [ ]

Dressing retention bandage (e.g. K-band) [ ]

Other [ ] If 'Other' please state: [ ]

4.8 Hosiery (stockings) for the leg affected by the selected wound: (***Please cross (x) one option*****).**

N/A [ ] Standard class 1 [ ]

Standard class 2 [ ] Standard class 3 [ ]

Made to measure Class 1 [ ] Made to measure Class 2 [ ]

Made to measure Class 3 [ ] Standard two layer compression hosiery (aiming to deliver 40mmhg) [ ]

Made to measure two layer compression hosiery (aiming to deliver 40mmhg) [ ]

Other [ ] If 'Other' please state: [ ]

For peer review only

Please record any equipment used relating to the selected wound:

4.9 Pressure relief and mobility: ***(please cross (x) all that apply)***

Pressure relieving	**mattresses:**	**cushions:**
High specification foam replacement e.g. MSS gilde	☐	☐
Air alternating replacement e.g. Quattro deep cell prime	☐	☐
Air alternating overlay e.g. Alpha excel	☐	☐
Repose (overlay system)	☐	☐
Foam overlay	☐	☐

Crutches ☐

Wheelchair ☐

Other ☐

If 'Other' please state:

4.10 Footwear/Orthotics/Foot pressure reliving equipment: ***(please cross (x) all that apply)***

Prescribed footwear ☐

Footwear adaptation ☐

Insoles ☐

Heel pressure relief ☐
e.g. Repose Heel Trough, PROFO boot

Other ☐ If 'Other' please state:

4.11 Other therapies for the selected wound: ***(please cross (x) all that apply)***

Hyperbaric oxygen therapy ☐

Physiotherapy ☐

Occupational therapy ☐

Negative pressure wound therapy ☐

Other ☐ If 'Other' please state:

For peer review only

4.12 Treatment objectives for the selected wound: ***(please cross (x) all that apply)***

Debridement ☐ Absorption (removal of exudate) ☐

Hydration (keep the wound moist) ☐ Microbial load management ☐

Odour Management ☐ Reduce overgranulation ☐

Encourage granulation ☐ Protection ☐

Other ☐ If 'other' please state: ☐

4.13 If the selected wound is a PRESSURE ULCER record its category here (otherwise leave blank). (*Please cross (x) one option).*

I ☐ II ☐ III ☐ IV ☐ Unstageable ☐ Not applicable ☐

If the selected wound is a LEG ULCER please record the following (otherwise leave blank):

4.14 Is a Doppler ABPI for the leg with the selected wound recorded in the notes? (*Please cross (x) one option).*

Yes ☐ No ☐ Don't know ☐

4.15 If yes – please note the ABPI measure here:

☐☐ • ☐☐

For peer review only
